# Phasic Firing and Coincidence Detection by Subthreshold Negative Feedback: Divisive or Subtractive or, Better, Both

**DOI:** 10.3389/fncom.2017.00003

**Published:** 2017-02-02

**Authors:** Gemma Huguet, Xiangying Meng, John Rinzel

**Affiliations:** ^1^Departament de Matemàtiques, Universitat Politècnica de CatalunyaBarcelona, Spain; ^2^Biology Department, University of MarylandCollege Park, MD, USA; ^3^Center for Neural Science, New York UniversityNew York, NY, USA; ^4^Courant Institute of Mathematical Sciences, New York UniversityNew York, NY, USA

**Keywords:** phasic firing, type III excitability, divisive, subtractive, coincidence detection, phase-locking

## Abstract

Phasic neurons typically fire only for a fast-rising input, say at the onset of a step current, but not for steady or slow inputs, a property associated with type III excitability. Phasic neurons can show extraordinary temporal precision for phase locking and coincidence detection. Exemplars are found in the auditory brain stem where precise timing is used in sound localization. Phasicness at the cellular level arises from a dynamic, voltage-gated, negative feedback that can be recruited subthreshold, preventing the neuron from reaching spike threshold if the voltage does not rise fast enough. We consider two mechanisms for phasicness: a low threshold potassium current (subtractive mechanism) and a sodium current with subthreshold inactivation (divisive mechanism). We develop and analyze three reduced models with either divisive or subtractive mechanisms or both to gain insight into the dynamical mechanisms for the potentially high temporal precision of type III-excitable neurons. We compare their firing properties and performance for a range of stimuli. The models have characteristic non-monotonic input-output relations, firing rate vs. input intensity, for either stochastic current injection or Poisson-timed excitatory synaptic conductance trains. We assess performance according to precision of phase-locking and coincidence detection by the models' responses to repetitive packets of unitary excitatory synaptic inputs with more or less temporal coherence. We find that each mechanism contributes features but best performance is attained if both are present. The subtractive mechanism confers extraordinary precision for phase locking and coincidence detection but only within a restricted parameter range when the divisive mechanism of sodium inactivation is inoperative. The divisive mechanism guarantees robustness of phasic properties, without compromising excitability, although with somewhat less precision. Finally, we demonstrate that brief transient inhibition if properly timed can enhance the reliability of firing.

## 1. Introduction

Phasic neurons may fire at the onset of a step input, typically once, but not during the steady portion and not for slowly varying inputs. This property of phasic firing is often called type III excitability, in contrast to repetitive firing for slow inputs for type I and II excitable neurons (Hodgkin, 1948; Svirskis et al., 2002; Izhikevich, 2007; Prescott et al., 2008a; Meng et al., 2012; Rinzel and Huguet, 2013). Phasic neurons can show extraordinary temporal precision for phase locking and coincidence detection. Some examples of phasic neurons include: auditory brain stem neurons that are involved with precise timing computations (Oertel, 1999; Schnupp and Carr, 2009; Carr and Macleod, 2010), some spinal cord neurons (Prescott and De Koninck, 2002; Prescott et al., 2008a), and even the squid giant axon (Clay et al., 2008).

Underlying phasicness is a dynamic, voltage-gated, negative feedback that can be recruited subthreshold, preventing the neuron from reaching spike threshold if an increasing input does not rise fast enough. Various cellular mechanisms can implement the dynamic negative feedback. Type III excitability may be due to an outward current (say, potassium K^+^) that activates relatively fast for subthreshold voltages (Rathouz and Trussell, 1998; Svirskis et al., 2002; Rothman and Manis, 2003; Prescott et al., 2008a). Thus, if an input current depolarizes a cell too slowly, the outward K^+^ current can activate and oppose the voltage rise, limiting the depolarization to subthreshold levels. In contrast, with rapid depolarization due to a fast-rising input current, the K^+^ current lags, allowing subsequent spike generation. During the spike, the K^+^ current strongly activates, precluding subsequent spikes, and therefore, repetitive firing.

This subtractive (K^+^ current) mechanism is not the only way to generate type III excitability. Indeed, a divisive mechanism, such as a fast-activating, but transient, inward (sodium Na^+^ or calcium) current with suitably recruitable inactivation, can also generate type III excitability (Prescott and De Koninck, 2002; Svirskis et al., 2004; Gai et al., 2009; Platkiewicz and Brette, 2011). The conceptual framework is the same. If the cell depolarizes slowly, the inactivation process develops before the inward current can activate and the cell will not fire a spike. If the depolarization is fast enough before inactivation of the inward current can occur, a spike (and only one spike) is produced.

In some auditory neurons both subtractive and divisive mechanisms contribute to phasic firing (Svirskis et al., 2004; Scott et al., 2010); it appears that inactivation of sodium current *I*_Na_ is *V*-gated at unusually low *V* values. Neurons in the auditory brain stem, where precise timing is important for sound localization (Oertel, 1983; Reyes et al., 1994; Oertel, 1999; Rothman and Manis, 2003), show extraordinary temporal precision (on sub-ms time scales) for phase locking and coincidence detection. One may wonder what is the contribution of each mechanism in shaping the properties of the system and what is gained by having two feedback processes.

To address these questions we utilize reduced 2 and 3-variable versions (Meng et al., 2012) of an 8-variable biophysically-based model developed by (Rothman and Manis, 2003). This 8-variable model, that we refer to as RM03, has been widely used for modeling the phasic firing of cells in the auditory brainstem. In RM03, the dominant mechanism for phasicness is a low-threshold potassium current (*I*_KLT_) (subtractive dominant mechanism). Phasic behavior is lost if the conductance of *I*_KLT_ is frozen at its resting value, but phasic firing can be restored with a divisive negative feedback mechanism by left-shifting the *V*-dependence of steady-state inactivation gating for sodium current. We characterize the features of the subtractive and divisive mechanisms by isolating each in our reduced models; one has only subtractive (S model), one only divisive (D model), and a third has a combination of both (C model).

Our reduced models permit mathematical analysis, including phase plane analysis, and thereby prediction and insight into the phasic firing properties. Using tools from bifurcation theory we can assess the range of conductances for which the neuron models show type III excitability. We observe that the subtractive mechanism if considered alone (i.e., if the *I*_Na_ is non-inactivating) requires a strong reduction of Na^+^ conductance in order to preserve its phasic properties and prevent a highly depolarized state for strong inputs (depolarized state of “lockup”). The presence of a divisive mechanism combined with a subtractive one, guarantees the robustness of the phasic properties of the system with respect to changes in channel density.

In this comparative study, we use a range of different stimuli that help us to elucidate the different contributions of each mechanism to the output properties. As a function of a steady input's amplitude the three models show no bifurcation to repetitive activity. Indeed, there is firing only in the presence of input fluctuations and firing probability shows strong sensitivity to input variance (Higgs et al., 2006; Lundstrom et al., 2008, 2009; Gai et al., 2009). In order to quantify this sensitivity, we compared the models' responses to ramps and periodic inputs, as well as their input-output curves (firing rate vs. mean input and varying noise strength, and firing rate vs. excitatory input frequency with random maximal conductance). We observe that the low-threshold K^+^ conductance in the S model prevents excessive depolarization, thus keeping the cell in the proper voltage operating range, and provides robustness to small input fluctuations. On the contrary, the D model has higher input resistance, thus more vulnerable to input fluctuations and mean depolarization, which ultimately leads to excitability loss from *I*_Na_ inactivation.

In order to assess coincidence-detection and phase-locking, we inject periodic trains of excitatory inputs, created from many small excitatory postsynaptic conductances (EPSGs) with random event times, that can be more or less synchronized. We vary the input frequency and vector strength (a measure of coincidence), and we compute the output firing rate (a measure of entrainment) and vector strength (a measure of phase locking). We find that while both mechanims show enhancement of vector strength, subtractive spikers can out-perform divisive spikers for temporal precision and coincidence detection, especially in the presence of a background noise. Finally, we explore the effects of background constant inhibition as well as timed inhibition and we observe that inhibition can have contrasting effects on the firing rate, positive or negative, but the D model shows less sensitivity to the arrival time of the inhibitory inputs compared to the S and C models, especially at high frequencies.

Our results suggest that phasic neurons equipped with two negative feedback processes are more robust to changes in applied currents and conductance densities than models that possess only one negative feedback mechanism, while they show stronger coincidence detection properties.

## 2. Methods

### 2.1. Neuron models

Rothman and Manis (2003) developed a Hodgkin-Huxley-like neuron model that has been widely used for modeling the phasic firing of cells in the auditory brainstem. It consists of a sodium current *I*_Na_, a high-threshold (*I*_KHT_) and a low-threshold (*I*_KLT_) potassium currents, a hyperpolarization-activated cation current *I*_*h*_ and a leak current *I*_lk_. The current balance equation has the following expression:

(1) $$C\frac{dV}{dt}=-I_{\text{Na}}-I_{\text{KHT}}-I_{\text{KLT}}-I_{\text{h}}-I_{\text{lk}}+I(t),$$

where *C* is the membrane capacitance, *V* is the membrane voltage and *I*(*t*) is the external input current. Each ionic current *I*_*i*_ is governed by an activation and/or inactivation variable, and Equation (1) can be written as

(2) $$\begin{array}{l} C\frac{dV}{dt}=2[-{\bar{g}}_{\text{Na}}m^{3}h(V-E_{\text{Na}})-{\bar{g}}_{\text{KHT}}(0.85n^{2}+0.15p)(V-E_{\text{KHT}})\;\\ \;-{\bar{g}}_{\text{KLT}}w^{4}z(V-E_{\text{K}})-{\bar{g}}_{h}r(V-E_{h})-{\bar{g}}_{\text{lk}}(V-E_{\text{lk}})]+I(t), \end{array}$$

where ḡ_*i*_ and *E*_*i*_ are respectively the maximal conductances and reversal potentials for the ionic current *i* = Na, KHT, KLT, h, lk. Maximal conductances and channel gating rates are multiplied by a factor of 2 and 3, respectively, as in Gai et al. (2009), to mimic the brain slices during whole cell recordings at temperature 32°C.

Of particular interest for our problem is the dynamics of activation of low-threshold potassium current *I*_KLT_ governed by the variable *w* and the dynamics of inactivation of the transient sodium current *I*_Na_ governed by the variable *h*.

To highlight the primary biophysical mechanisms for phasic firing properties and to facilitate our analysis, we proceed as in Meng et al. (2012), and develop 3 reduced versions of the 8-variable RM03 model by identifying and approximating some nonessential features for the model's excitability and spike generation mechanism. Namely, we set *m* = *m*_∞_(*V*), we freeze the inactivation gating variable *z* of *I*_KLT_ and the activation gating variable *r* of *I*_*h*_, and we remove *I*_KLT_ (see Meng et al., 2012 for more details and a justification of this simplification).

The reduced models considered are two 2-variable models, both divisive and subtractive dominant versions, and a third 3-dimensional model that combines both mechanisms.

#### 2.1.1. Subtractive (S) model

In order to isolate the subtractive mechanism, we disable the subthreshold dynamic negative feedback provided by inactivation of *I*_Na_ by freezing the inactivation variable *h* at its value at the resting state for the other two models. To do so and still retain the type III excitability property, we need to reduce ḡ_Na_ (see Appendix B in Supplementary Material). The model then writes as:

(3) $$\begin{array}{l} C\frac{dV}{dt}=-2({\bar{g}}_{\text{Na}}m_{\infty }(V^{3})h_{0}(V-E_{\text{Na}})+{\bar{g}}_{\text{KLT}}w^{4}z_{0}(V-E_{\text{K}})\\ \;+g_{1}(V-E_{1}))+I\\ \frac{dw}{dt}=3\frac{w_{\infty }(V)-w}{\tau_{w}(V)}, \end{array}$$

where *C* = 12pF, ḡ_Na_ = 177nS, *h*_0_ = 0.22, ḡ_KLT_ = 200nS, *z*_0_ = 0.662, *g*_*l*_ = 4.97nS, *E*_*Na*_ = 55mV, *E*_*K*_ = −70mV and *E*_*l*_ = −52.024mV. Notice that we obtained *g*_l_ and *E*_l_ from the original RM03 model by setting *g*_l_ = ḡ_h_r_0_+ḡ_lk_ and *E*_l_ = 1/*g*_l_(ḡ_h_*r*_0_*E*_h_ + ḡ_lk_*E*_lk_).

**Figure 1:**
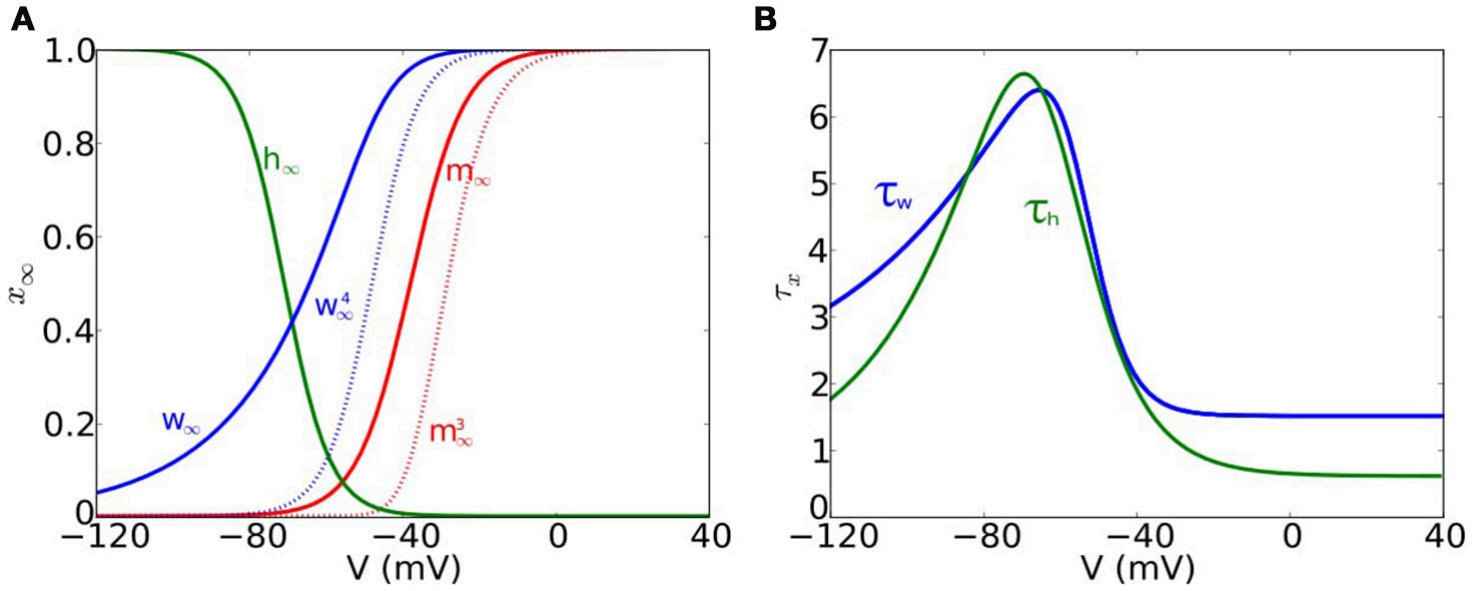
**Voltage-dependent functions of the models**. **(A)** Voltage-dependent steady-state functions for the gating variables of the ionic currents: sodium activation *m*_∞_ (solid red), sodium inactivation *h*_∞_ (green) and potassium activation *w*_∞_ (solid blue). We also include $w_{\infty }^{4}$ (dotted blue) and $m_{\infty }^{3}$ (dotted red). **(B)** Time constant functions for sodium inactivation τ_*h*_ (green) and potassium activation τ_*w*_ (blue).

The steady-state functions *m*_∞_(*V*) and *w*_∞_(*V*) (see Figure 1A) are given by

$$m_{\infty }\left(V\right)={\left(1+e^{-(V+38)/7}\right)}^{-1},$$

and

(4) $$w_{\infty }\left(V\right)={\left(1+e^{(-(V+48)/6}\right)}^{-1/4},$$

respectively, and the time function for *w* (see Figure 1B) is

(5) $$\tau_{w}\left(V\right)=1.5+\frac{100}{6e^{(V+60)/6}+16e^{-(V+60)/45}}.$$

#### 2.1.2. Divisive (D) model

We can disable the subthreshold dynamic negative feedback provided by activation of *I*_KLT_ by freezing the conductance *g*_KLT_ (activation and inactivation variables *w* and *z*) to its value at the resting state. In this case, the only subthrehsold dynamic negative feedback that is left in the model is sodium inactivation, but in order to ensure phasic firing for a steady input, we manipulated the model so that sodium inactivation is shifted to lower values of voltage (i.e., *h*_∞_(*V* + 6)). The model then reduces to:

(6) $$\begin{array}{l} C\frac{dV}{dt}=-2({\bar{g}}_{\text{Na}}m_{\infty }{\left(V\right)}^{3}h(V-E_{\text{Na}})+{\bar{g}}_{\text{KLT}}w_{0}^{4}z_{0}(V-E_{\text{K}})\\ +g_{1}(V-E_{1}))+I\\ \frac{dw}{dt}=3\frac{h_{\infty }(V)-h}{\tau_{h}(V)}, \end{array}$$

where *w*_0_ = 0.512, ḡ_Na_ = 500 nS and the other parameter values are as in the S model.

The steady-state function *h*_∞_(*V*) (see Figure 1A) is given by

(7) $$h_{\infty }\left(V\right)={\left(1+e^{(V+h_{shift}+65)/6}\right)}^{-1},$$

and the time function for *h* (see Figure 1B) is

(8) $$\tau_{h}(V)=\frac{100}{7e^{(V+60+h_{shift})/11}+10e^{-(V+60+h_{shift})/15}}+0.6,$$

with *h*_*shift*_ = 6.

#### 2.1.3. Combined (C) model

The model has both dynamic subthreshold negative feedback mechanisms: activation of *I*_KLT_ (as modeled for the S model) and inactivation of *I*_Na_ (as modeled for the D model). The voltage equation is given by:

(9) $$\begin{array}{l} C\frac{dV}{dt}=-2({\bar{g}}_{\text{Na}}m_{\infty }{\left(V\right)}^{3}h(V-E_{\text{Na}})+\;{\bar{g}}_{\text{KLT}}w^{4}z_{0}(V-\;E_{\text{K}})\\ \;\;\;\;\;\;\;\;\;\;\;\;\;+g_{l}\;(V-E_{l}))+I\\ \frac{dw}{dt}=3\frac{w_{\infty }(V)-w}{\tau_{w}(V)}\\ \frac{dh}{dt}=3\frac{h_{\infty }(V)-h}{\tau_{h}(V)}, \end{array}$$

where ḡ_Na_ = 500 and the other parameter values are as before. The functions *h*_∞_(*V*) and τ_*h*_ are given in Equations (7) and (8), respectively (as in the D model), and *w*_∞_ and τ_*w*_ are given in Equations (4) and (5), respectively (as in the S model).

### 2.2. Time scales ratio

We compute the ratio between voltage and gating variable time constants for the divisive and the subtractive models.

We define the total conductance as

$$g_{tot}(V,h,w)=2(g_{\text{Na}}(V,h)+g_{\text{KLT}}(V,w)+g_{l}).$$

The ratio for the divisive model is given by:

$$r(V,h)=\frac{3C}{g_{tot}(V,h,w_{0})\tau_{h}(V)},$$

and for the subtractive:

$$r(V,w)=\frac{3C}{g_{tot}(V,h_{0},w)\tau_{w}(V)}.$$

Notice that values of *r* close to 1 indicate that the two variables evolve on a similar time scale, while values of *r* close to 0 indicate that the voltage evolves on a much faster time-scale than the gating variable.

### 2.3. Inputs

We consider several types of deterministic inputs: steps, ramps and also a half-wave rectified sinusoidal input *I*(*t*) with amplitude *A* and frequency ω,

(10) $$I(t)=A{\left[\sin(2\pi \omega t)\right]}^{+},$$

where [·]^+^ = max(·, 0). In some cases we inject external noisy input of the form *I*_*noise*_ = *C*ση(*t*), where η(*t*) is a “white noise” process.

We also consider conductance based synaptic-like currents. Each synaptic site generates a minimal excitatory or inhibitory postsynaptic conductance (EPSG/IPSG) that is modeled as an alpha function with time constant τ_syn_ = 0.3 ms, unless otherwise stated, and maximal conductance *g*_*max*_:

(11) $$g_{syn}(t)=g_{max}\frac{t}{\tau_{syn}}e^{1-t/\tau_{syn}}.$$

Thus, conductance-based synaptic currents have the form:

$$I_{syn}(t)=g_{syn}(t-t_{s})(V-E_{syn}),$$

for *t* ≥ *t*_*s*_, where *E*_*syn*_ = 0 and *E*_*syn*_ = −75 mV for excitatory and inhibitory synaptic inputs, respectively, and *t*_*s*_ is the pre-synaptic spike time.

For the case of steady (but random) synaptic inputs, the synaptic event times are Poisson distributed with a given frequency and their maximal conductances are modeled as a summation of *N* = 7 independent miniature EPSGs with fixed amplitude, each of which occurs with probability *p* = 1/2. Thus, maximal conductances for composite EPSGs are discrete and modeled with a binomial distribution, *B*(*N, p*), where *N* = 7 is the maximum number of mEPSGs each with probability *p* = 0.5 of occurence. Thus, the mean number of mEPGS is 3.5, i.e., there will be 50% probability for *N* ≤ 3 and 50% probability for *N* ≥ 4.

In order to test coincidence detection, we inject periodically modulated excitatory synaptic inputs from several fibers/sites as in Jercog et al. (2010), similar to what might occur *in vivo* (Joris et al., 1994). Each cycle's composite input was generated from eight small (mini) excitatory postsynaptic conductances (mEPSGs) modeled as alpha functions with fixed amplitude and event times per site drawn from a von Mises distribution independently at each cycle. The von Mises probability density function for the angle θ is given by:

(12) $$f(\theta ,a,b)=\frac{e^{b\cos(\theta -a)}}{2\pi I_{0}(b)},$$

where *a* = 1/4 is the mean and *b* is the temporal coherence (*b* = 0 corresponds to a uniform distribution and as *b* increases the distribution becomes more concentrated about the angle *a*), and *I*_0_(*x*) is the modified Bessel function of order 0. Notice that to vary *b* is equivalent to vary the vector strength of the periodic input train. The vector strength (VS), also known as “synchronization index” (Goldberg and Brown, 1969), is a measure of how clustered are events over a cycle. To compute the VS one associates to each event time a vector on the unit circle with a phase angle and computes the mean vector. The VS is given by the length of the mean vector. Perfect clustering is obtained when *VS* = 1. The relationship between VS and the temporal coherence *b* of the input distribution is given by:

$$VS(b)=I_{1}(b)/I_{0}(b),$$

where *I*_0_(*x*) and *I*_1_(*x*) are the modified Bessel function of order 0 and 1, respectively.

In our simulations we chose maximal conductances for individual mEPSGs according to the following criteria: for low input strength, we pick *g*_*max,e*_ so that six coincident inputs, but not less, generate a composite EPSG that exceeds threshold for spike at rest, and for high input strength we reduce this number to four coincident inputs. Notice, that since the EPSG threshold for spike is different for each model, we used different values of *g*_*max,e*_ for each model.

In order to test the role of timed inhibition we also inject periodic inhibitory inputs generated in the same way as the excitatory ones: composite IPSGs are obtained from mini-IPSGs (mIPSGs) modeled as alpha functions (see Equation 11) with maximal conductance *g*_*max,i*_ and the timings in the arrival of mIPSGs are also drawn from a von Mises distribution. The frequency of the inhibitory input will be the same as for the excitatory input, but we will vary other parameters for inhibition: the temporal coherence *b*_*i*_ in the von Mises distribution (Equation 12), the maximal conductance *g*_*max,i*_ and time scale τ_*i*_ of the mIPSGs (Equation 11). Since we want explore the role that arrival time of inhibition plays in the maximal response of the system, we also vary the phase difference φ between the inhibitory and the excitatory inputs (φ = *a*_*i*_ − *a*_*e*_ (mod 1), φ ∈ [0, 1]), where *a*_*i*_ and *a*_*e*_ are the means of the von Mises distribution for the inhibitory and the excitatory input trains, respectively. Notice that when φ is close to 0, inhibition just follows excitation, while when φ is close to 1, inhibition just precedes excitation.

### 2.4. Criterion for spike identification or detection

**Figure 2:**
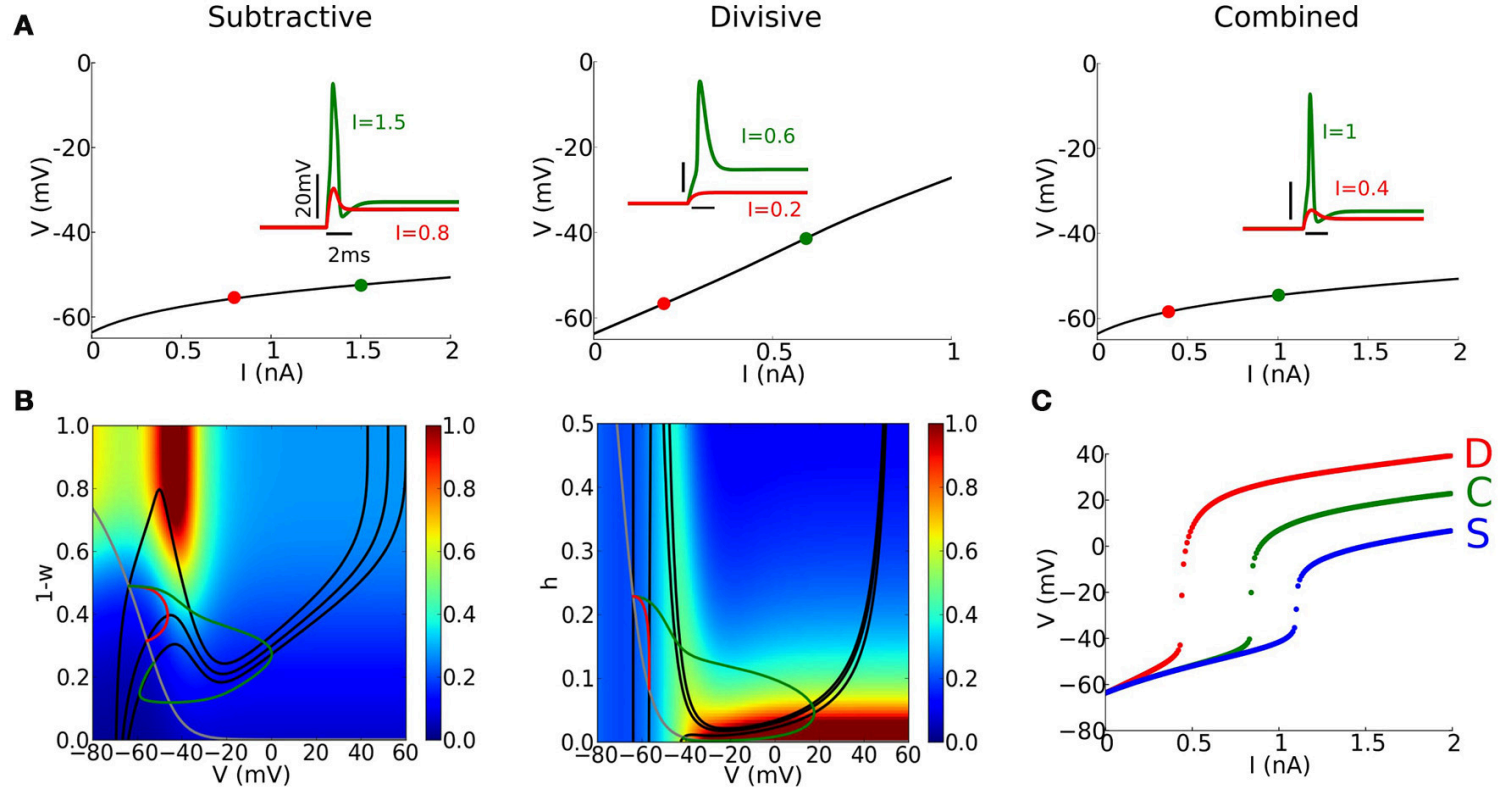
**Basic properties of phasic firing**. **(A)** Steady state (fixed point) continuation for S (left), D (center), and C (right) models for a steady current *I*. Stable fixed point (black solid) does not destabilize. (Inset) Voltage responses to a step current of different amplitudes indicated in each panel. **(B)** Phase-plane portraits and trajectories corresponding to the same step current as in **(A)** for S and D models. (Left, S model) *V*-nullclines for *I* = 0, 0.8, 1.5 nA (solid black curve) and *w*-nullcline (gray curve). Colors indicate ratio between *V* and *w* time-scales considering only ratios smaller than 1. (Middle, D model) *V*-nullclines for *I* = 0, 0.2, 0.6 nA (solid black curve) and *h*-nullcline (gray curve). Colors indicate ratio between *V* and *h* time-scales considering only ratios smaller than 1. **(C)** Maximal value of *V* in response to a step current of varying amplitude (*x*-axis) for S (blue), D (red), and C (green) models.

RM03-like models have low input resistance. The high conductance shunts EPSCs as well as spike currents. Strong EPSGs are required to elicit a spike and distinguishing spikes from EPSPs requires care. Although spikes may vary in amplitude, we observe that there is a sharp rise in voltage response when the input exceeds a certain value (see Figure 2C), showing the presence of a regenerative process. Thus, our criterion for spike detection requires that *V* passes a set level (−20 mV) and that the net intrinsic current when *V* = −20 mV is negative, i.e., dominated by sodium current (in order to detect the regenerative process).

### 2.5. Simulation methods

Equations were integrated numerically in C using an implicit 4th order Runge-Kutta method. Stochastic differential equations had a white noise term and were integrated using an Euler-Maruyama method for stochastic differential equations (Higham, 2001) with time step Δ*t* = 0.005 ms, along with a random number generator from the GSL-GNU Scientific Library. The bifurcation diagrams were computed using the Auto feature in XPPAUT. We used Matlab and Python to analyze and plot the data.

## 3. Results

### 3.1. Reduced models with divisive or subtractive mechanisms for type III excitability

We have developed reduced models (with 2 or 3 variables) that show type III excitability (each model fires once only at the onset of a step current and not repetitively thereafter) using two different biological mechanisms, subtractive and divisive, that we managed to isolate.

Our starting point model is a reduced version of the Rothman and Manis model (RM03) (Rothman and Manis, 2003) for phasic neurons in the auditory brainstem (see Equation 1 in Methods), that we previously showed behaves semi-quantitatively and qualitatively as the full model (Meng et al., 2012). This reduced version contains a potassium current that is partially activated at *V*_*rest*_ and can significantly activate for subthreshold voltages, and a transient sodium current that can significantly inactivate for subthreshold voltages. Noting that “threshold” does not correspond to a fixed voltage value for spike initiation, especially in the case of phasic models (Azouz and Gray, 2000; Platkiewicz and Brette, 2010, 2011), we use as threshold the voltage value at which sodium activation (responsible of spike generation) rises sharply (i.e., $m_{\infty }^{3}\left(V\right)$), which is approximately −40 mV. This voltage value would correspond to spike initiation if the dynamic subthreshold negative feedback factors were not present (see Section Methods for our precise criterion for spike identification). For this reduced RM03 model we also remove the *I*_KLT_ current and freeze the activation variable of *I*_*h*_, as well as the *I*_KLT_ inactivation variable at their resting values (*V*_*rest*_ = −63.6 mV).

Now, we formulate 2-variable models that isolate the two negative feedback mechanisms. The Substractive (S) model (see Equation 3) isolates *I*_KLT_ using a non-inactivating sodium current by freezing the inactivation gating variable of the RM03 model at its resting state value. Without dynamic Na^+^ inactivation, the S model can develop bistability (coexistence of *V*_*rest*_ with a stable depolarized state). To prevent such bistability and preserve type III excitability we reduce ḡ_Na_ from 1000 to 177 nS (see Appendix B in Supplementary Material for more details). Although this is a strong reduction (by a factor of 1/5 or so), notice that Na^+^ strongly inactivates at subthreshold values of voltage and spikes ocurr with *h* approximately 0.1, thus the total Na^+^ conductance at spike onset is more comparable. Nevertheless, even with this reduction, the density of sodium channels for the *S* model is sufficient to amplify subthreshold synaptic potentials, thus retaining the excitability property. The divisive (D) model (see Equation 6) isolates Na^+^ inactivation by freezing the potassium activation variable at its resting value (*w*_0_ = *w*_∞_(*V*_*rest*_)). However, freezing *w* to its resting value converts this reduced D model (as well as the original RM03) from phasic to tonic (Day et al., 2008; Gai et al., 2009, 2010; Meng et al., 2012). To counteract this effect and retain phasic firing in the D model we shift *h*_∞_ by 6mV leftwards with respect to the original *h*_∞_ in RM03 (see Figure 1). Moreover, in order to better compare the results with the S model we decrease ḡ_Na_ from 1000nS to 500nS. Finally, we combine these two mechanisms in a 3-variable model: the combined (C) model (see Equation 9) has *I*_KLT_ of the S model and *I*_Na_ of the D model.

### 3.2. Dynamic properties for different type III mechanisms

Each of the models, C, D, S, fires phasically (only one spike, if any, at the onset of the input; see Figure 2A, inset) and its steady state *V*_*ss*_ depends on *I*, the applied current, but is stable for any *I* (Figure 2A). There is no bifurcaton to repetitive firing on the *V*_*ss*_ vs. *I* curve - a signature of type III excitability. For a slow modulation of the input current *I* there is no spiking; the voltage tracks the *V*_*ss*_ vs. *I* relation. Depolarization in the S and C models with *I* is modest because of *I*_KLT_ activation. In contrast to this rectification in the *V*_*ss*_ vs. *I* relation for S and C, the D model has a nearly linear relation without *V*-gating of *I*_KLT_ and negligible steady state *I*_Na_. Thus, the set of voltages at steady state spans a wider range for the D model than for the S and C models.

In a phasic firing system, spikes are elicited only for transient inputs, fast enough and strong enough inputs. For step current input our models respond sensitively to a step's amplitude (Figures 2A(inset),**C**), effectively showing a threshold-like behavior for this stimulus class. The C model has higher current threshold than the D model – they have the same ḡ_Na_ but C has two negative feedback processes. S has the highest current threshold, reflecting the decreased ḡ_Na_. Notice that the amplitude of spikes is modulated accordingly.

Phase plane analysis and relative time scales between voltage and gating variable help us understand dynamic features of spike generation (see Figure 2B and Methods Section). The trajectories corresponding to the time courses in Figure 2A (inset) reveal the characteristics of excitability: amplification of *V* for an adequate stimulus and then recruitment of dynamic negative feedback (Figure 2B). For these phasic systems, the steady state is always on the “left branch” for each *I*-value, guaranteeing its stability (Rinzel and Huguet, 2013). For steady strong input the *V*-nullcline's cubic or N-shape is greatly diminished corresponding to suppressed excitability (strongly activated *I*_KLT_ or strongly inactivated *I*_Na_). Notice that this change is more dramatic in the D model than in the S model (the *V*-nullcline for *I* = 0.6 nA in the D model is hardly visible).

When the external input *I* increases, the steady state drifts downward along the left branch and the phase point approaches the steady state. In the S model, activation of *I*_KLT_ precludes excessive depolarization, limiting the steady state voltage to values no higher than −60 to −50 mV. Close to the resting state, the voltage and the gating variable have “comparable” time-scales (within a factor of 3), and the phase point does not escape rightward directly (red trajectories in Figure 2B). Only for large enough input *I*, the phase point can enter the region with a clear time-scale separation (blue region for values of *V* above −40 mV), and escape rightward toward the right branch of the *V*-nullcline, generating a spike (green trajectories in Figure 2B). Repolarization of the membrane potential after a spike is only due to the Ohmic leak current in the D model, unlike S and C models that have the *V*-gated potassium current, and there is no hyperpolarization when the spike terminates.

For a time-varying input the *V*-nullcline and target steady state move with the stimulus. Thus, for a slow stimulus ramp, the phase point tracks slowly the drifting steady state and there is no spike. For a fast enough ramp the phase point can escape rightward and lead to a single spike and then settle to a stable depolarized steady state. For this reason, phasic neurons are often called differentiators because they respond to fast-rising, but not to slow-rising, inputs (Ferragamo and Oertel, 2002; Svirskis et al., 2002; McGinley and Oertel, 2006; Izhikevich, 2007; Ratte et al., 2014). Our D model is a less sensitive differentiator; its critical value for the ramp slope to elicit an action potential is lower (see Figure S1A) and, in response to a half-wave rectified sinusoidal input (Equation 10), it can fire at lower frequencies than C and S (see Figure S1B).

### 3.3. Type III excitability is more robust in the presence of Na^+^ inactivation

**Figure 3:**
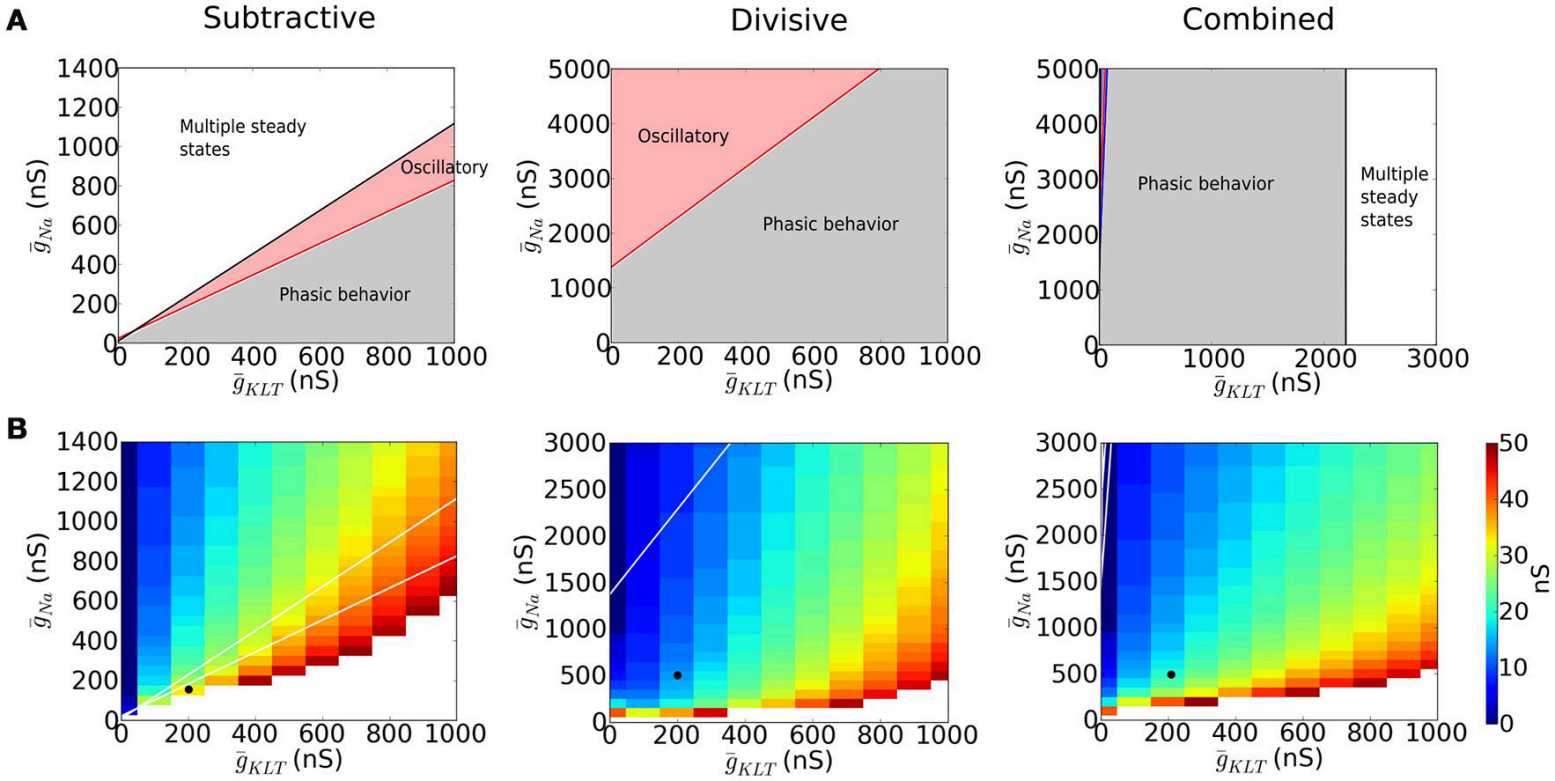
**Dynamics of models with varying maximal sodium (ḡ_Na_) and potassium (ḡ_KLT_) conductances**. **(A)** Different dynamic regimes are indicated with different colors. In the gray area the fixed point is stable for the biologically plausible range of voltages *V* ∈ (−100, 40) (see Appendix A in Supplementary Material for more details) and no bifurcation occurs (type III excitability). In the red region, a Hopf bifurcation occurs (type II excitability). The white area has saddle-node bifurcations of the fixed points and multiple steady states are possible. **(B)** Minimal EPSG strength that produces a spike for an EPSG of the form $g_{ex}\left(t\right)=g_{max}\left(t/0.3\right)e^{1-t/0.3}$, assuming that the neuron is at its resting state. White area corresponds to no spikes. The black dot indicates the maximal conductances chosen for each model to perform the comparative study.

Type III excitability occurs in each of our representative models but more or less robustly for mixtures of *I*_Na_ and *I*_KLT_. We identified regions in the parameter space (ḡ_Na_, ḡ_KLT_) where the models behave phasically, i.e. the fixed point is stable for any value of *I* (Figure 3A, gray region). For other ḡ_Na_−ḡ_KLT_ combinations the models show destabilization of the fixed point through a Hopf bifurcation and repetitive firing (Figure 3A, red region) and the possiblity of multiple steady states, including a stable steady state of high voltage, “lockup,” (Figure 3A, white region).

The divisive mechanism is key to guarantee the robustness of the phasic properties of the system with respect to changes in channel density. Removal of Na^+^ inactivation, as in the S model, strongly constrains the parameter range for type III excitability. To avoid “lockup” in the S model (large white region in Figure 3A) we chose its value of ḡ_Na_ smaller than for D and C models; consequently, a stronger input was required for the S model to be excited (see Figure 2C). By comparing the parameter spaces of the D and C models, we observe that when ḡ_Na_ in the D model is increased, the system transitions to a regime with oscillatory behavior, while the presence of *I*_KLT_ narrows this region (compare Figure 3A for D and C).

The threshold for eliciting a spike with a brief synaptic excitatory conductance input (EPSG) depends on ḡ_Na_ and ḡ_KLT_ (see Figure 3B). If ḡ_Na_ is small, then the system is not excitable (white region in Figure 3B). Of course, as ḡ_Na_ increases the system becomes more excitable and the EPSG threshold decreases. Moreover, if ḡ_KLT_ is large, more inward current (*I*_Na_ plus the excitatory current) is needed to overcome the opposing potassium current and EPSG threshold increases. Thus, in Figure 3B, along contours, ḡ_Na_ increases as ḡ_KLT_ increases.

### 3.4. Non-monotonic *f*-*I* curves for noisy, constant mean input current

Neurons and models with type III excitability respond to input transients but not to slowly varying inputs. In contrast, type I and type II excitable systems fire repetitively for steady input current. Their input-output relations, firing frequency vs. current (*f*-*I* curve), typically show monotonic increase over much of the *I*-range and then at high *I*-values loss of oscillation with either gradually decreasing amplitude or with a sudden drop in frequency and amplitude (Rinzel and Ermentrout, 1998; Borisyuk and Rinzel, 2005; Rinzel and Huguet, 2013). For type III excitability, an *f*-*I* relation quantifies the response to a fluctuating input: the mean firing frequency vs. the mean of a noisy current. The *f*-*I* curve is non-monotonic, as seen in some illustrative cases (Higgs et al., 2006; Lundstrom et al., 2008, 2009; Gai et al., 2009), and particularly distinctive from typical (noise free) type I and II cases. The firing frequency (firing probability per unit time) decreases smoothly toward zero for high mean *I* and the *f*-*I* relation shows strong sensitivity to input variance.

**Figure 4:**
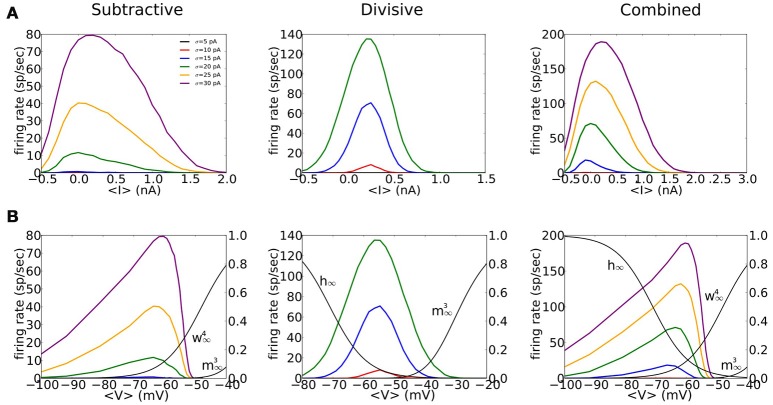
**Firing rate for noisy input current, as a function of mean input and mean voltage shows non-monotonic behavior**. **(A,B)** Inputs to the S (left), D (middle), and C (right) models are noisy currents modeled as Gaussian white noise with constant nonzero mean 〈*I*〉 and noise intensity σ = 5, 10, 15, 20, 25, 30 pA (different colors shown in legend). **(A)** Output firing rates vs. mean input current 〈*I*〉. **(B)** Output firing rates vs. mean voltage 〈*V*〉. Solid black thin curves are *h*_∞_, $m_{\infty }^{3}$, and $w_{\infty }^{4}$ used for each model. Mean voltage 〈*V*〉 are computed from the time series by ignoring a 4 ms time window that surrounds a spike.

Our three phasic models have non-monotonic *f*-*I* relations and show strong sensitivity to noise (Figure 4A). The *f*-*I* curves are similarly shaped for the S and C models but less symmetric than D's *f*-*I* curves. There is no repetitive firing without noise; the increase of firing probability with noise level evidences the models' sensitivity to input transients (different colors in Figure 4A). The S model fires less frequently than D or C to a given noise level, partly because ḡ_Na_ is smaller for the S model requiring stronger fluctuations to generate spikes (see Figure 3B). The C model shows intermediate sensitivity to noise.

We highlight the different effects of subthreshold *V*-dependent conductance gating by replotting the input-output relations of Figure 4A in terms of mean voltage, 〈*V*〉 (Figure 4B). For S and C the maximum firing rate occurs for 〈*V*〉 around *V*_*rest*_, slightly depolarized for D. The firing rate of S and C decreases for increased 〈*V*〉 and falls abruptly to zero for 〈*V*〉 just below −50 mV, less than the activation voltage for *I*_Na_. This abrupt falloff reflects the shunting effect of strongly activated *g*_KLT_ and reduced amplitude of *V* fluctuations. On the other hand, firing rate rises gradually with 〈*V*〉 below *V*_*rest*_ with substantial firing probability 〈*V*〉 well below *V*_*rest*_. At these hyperpolarized levels the input resistance is high (*g*_KLT_ is deactivated) and *V*-fluctuations are substantial. In contrast to the asymmetry in the *f*-*V* relations for S and C the relation for D is more symmetric, like that for *f*-*I* relation of D. Ohmic leak is the only conductance at the foot and tail of the *V*-range for D. Therefore, the input resistance and voltage fluctuations are comparable.

### 3.5. Non-monotonic input-output curves for stochastic synaptic input

**Figure 5:**
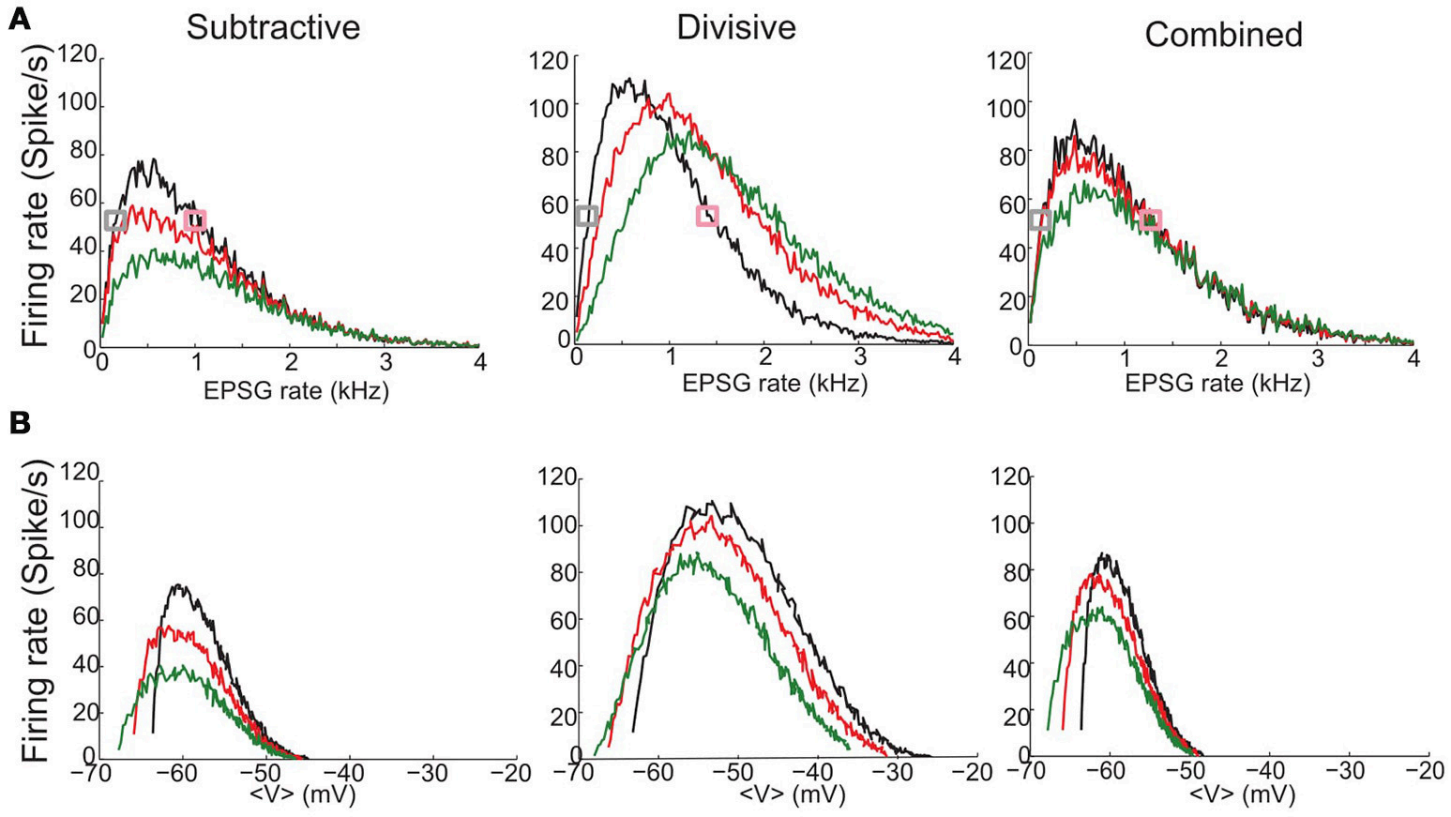
**Firing rate for stochastic synaptic inputs as a function of EPSG input rate and mean voltage depolarization shows different responses for constant inhibitory synaptic inputs**. **(A,B)** Inputs to the S (left), D (middle), and C (right) models are trains of excitatory synaptic conductance inputs (EPSGs) that are Poisson distributed in time and binomially-distributed in amplitude (see Section Methods). The mean amplitude is different for each model and is chosen according to the threshold to elicit a spike from the resting state: 8 nS (S model); 3.86 nS (D model) and 5.7 nS (C model). **(A)** Output firing rate as a fucntion of EPSG input rate (*x*-axis) in the absence of inhibition (black) and under the presence of a synaptic inhibitory current of constant conductance: *g*_inh_ = 10 (red), 20 nS (green). **(B)** Output firing rate obtained in **(A)** plotted a function of mean membrane potential 〈*V*〉. Mean voltages 〈*V*〉 are computed from the time series by ignoring a 4 ms time window that surrounds a spike. Curves are colored according to the inhibition strength as in **(A)**. Figure S3 shows output firing rate for a larger range of inhibitory strengths. Figure S2 shows spike triggered averages for the simulation runs indicated with gray and pink squares in **(A)**.

The noisy current input of Section 3.4 may be viewed as an idealization of random synaptic input delivered to a cell. Here, we consider the response to trains of excitatory synaptic conductance inputs (EPSGs). The event times are Poisson distributed and the EPSG amplitudes are binomially-distributed (see Section Methods) with mean amplitude that will just elicit a spike from the resting state. The three models show non-monotonic input-output curves, firing rate vs. EPSG input rate (Figure 5A, black curves), qualitatively as seen in Figure 4A for noisy current injection but some features differ. On the low input side the models fire for current injection (Figure 4A) even for non-positive 〈*I*〉, since by chance some positive current fluctuations are strong enough to cause a spike. But for synaptic input, firing only occurs for positive EPSG rate (Figure 5A). This feature is also seen in Figure 5B (black curves) where the firing rate for stochastic EPSG input drops abruptly to zero as 〈*V*〉 decreases below *V*_*rest*_; this corresponds to zero EPSG rate, i.e. no synaptic input. Substantial mean input, either noisy current injection or stochastic ESPGs, leads to depolarization and reduced firing probability. But for synaptic input, high EPSG rate leads to increased membrane conductance, thus EPSGs that might be superthreshold for lower input rates are not for higher rates; the EPSG threshold has effectively increased thereby attenuating the effect of input variance and smoothing the drop in firing rate (see more details in Figure S2). For the D model, the conductance nature of synaptic input accounts for the loss of symmetry in its input-output relation.

Synaptic inhibition differently affects our three models while they are undergoing stochastic EPSG input. For the S and C models, not surprisingly (although, see below), inhibition decreases the firing rate for a given EPSG rate (Figure 5A, red and green curves). In contrast, for the D model the relationship between firing rate and inhibition can be non-monotonic (Figure 5A), depending upon the EPSG rate. Specifically, the firing rate decreases with *g*_inh_ for low EPSG rate, whereas for high EPSG rate firing rate increases. Consequently, at moderate input rates the firing rate behaves non-monotonically, increasing then decreasing with *g*_inh_ (see more details in Figure S3).

There are some notable features of the input-output relations, firing rate vs. 〈*V*〉 (Figure 5B). Overall, the relations appear qualitatively similar for the 3 models, non-monotonic with a sharp rise and gradual fall for increasing 〈*V*〉. The “left branch” extends to lower 〈*V*〉 with *g*_inh_; this is because *g*_inh_ lowers the effective resting voltage, where firing rate descends to zero as 〈EPSG〉 rate decreases to zero. For the S and C models, in case of either stochastic EPSGs, here (Figure 5B), or for noisy current injection (Figure 4B), firing rate decreases from its maximum to near zero for the same 10 mV range: (−60, −50 mV). Notably, the maximum firing rate occurs at the same mean voltage independently of the inhibition level; this “sweet spot“ for firing is again located about the same voltages as for the noisy input (Figure 4B): ~−60mV for S and C and at a higher depolarized state (~−55mV) for D. From the perspective of a given 〈*V*〉 we see (except for 〈*V*〉 less than, say, −60 mV) that the firing rate decreases with *g*_inh_. The effect is monotonic decreasing in each model. The reason being that in order to maintain a fixed 〈*V*〉 as *g*_inh_ increases, the excitatory input, EPSG rate, must also increase. That is, to keep a balanced state of fixed 〈*V*〉 both synaptic excitation and inhibition increase or decrease together. The amount of increased *g*_*inh*_ that compensates for increased excitatory input to maintain a balanced 〈*V*〉 is smaller for the D model than for S and C due to D's larger input resistance (see Figure S3).

### 3.6. Precision and detection of coincident inputs for periodic drive

#### 3.6.1. Coincidence detection, reliability, and precision of entrainment

Neuron models with type III excitability are good coincidence detectors, as are neurons in the auditory brainstem. That is, they respond when multiple inputs arrive tightly-timed in a volley but not if the same inputs are dispersed in time. Moreover, such neurons can phase lock with high precision to an external periodic stimulus; they fire precisely timed on each stimulus cycle, sometimes enhancing the input precision (Joris et al., 1994; Joris and Smith, 2008).

**Figure 6:**
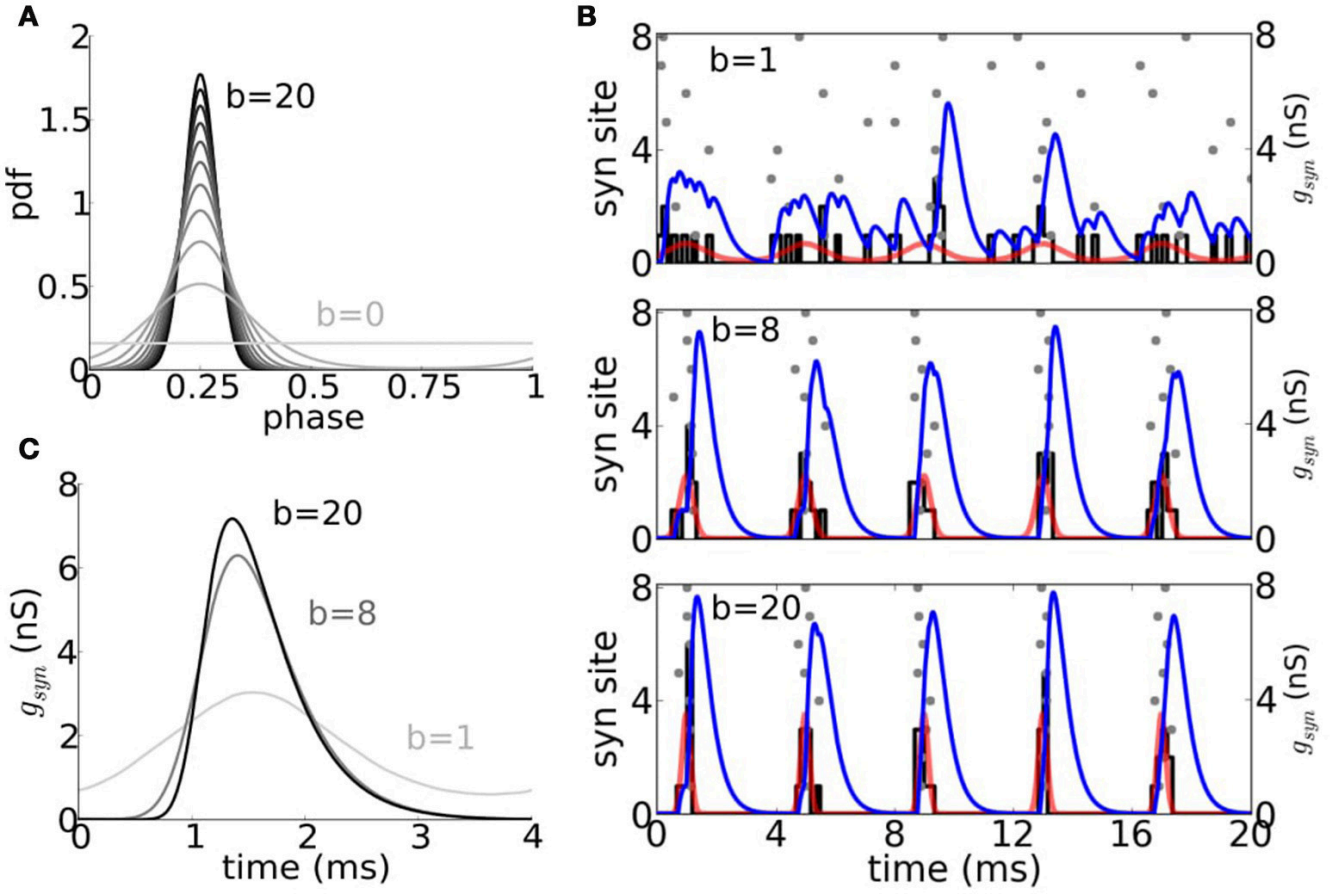
**Periodic trains of composite excitatory synaptic inputs with random event times**. **(A)** Probability density function (pdf) of the von Mises distribution for different values of the temporal coherence parameter *b* ranging between 0 and 20 in increments of 2 units. **(B)** Raster plot of event times per site (8 sites) drawn from a von Mises distribution (pdf shown in red) with *b* = 1 (top), *b* = 8 (middle), and *b* = 20 (bottom), together with the input period histogram (black) and composite EPSG (blue curve) obtained from the summation of individual mEPSG with *g*_*max*_ = 1 and *f* = 250 Hz (see Section Methods). **(C)** Averaged composite EPGS for *b* = 1, *b* = 8, and *b* = 20.

**Figure 7:**
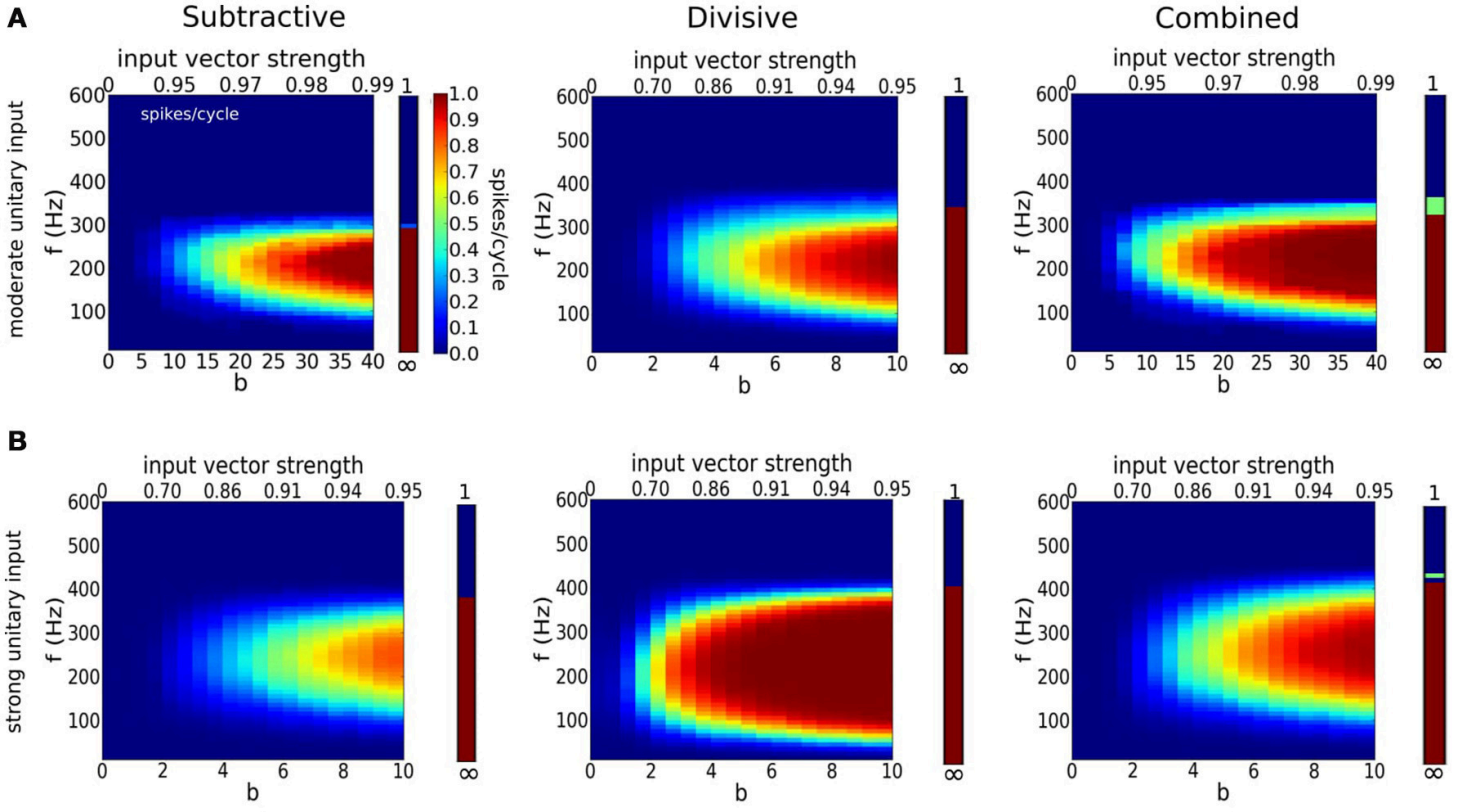
**Detection of coincident inputs for a periodic train of multi-synaptic inputs with varying temporal coherence and frequency**. **(A,B)** Firing probability per cycle (0 <spikes/cycle< 1) for S (left), D (middle), and C (right) models as a function of temporal coherence in the arrival of inputs (*x*-axis; bottom axis shows temporal coherence parameter *b* in von Mises distribution, while top axis shows its corresponding input vector strength) and input frequency *f* (*y*-axis). Bars on the right hand side of panels correspond to firing rate when dispersion is null (*b* = ∞). Notice that temporal coherence *b* refers to a cycle, thus the dispersion of inputs in absolute time for a given value of *b* decreases with input frequency. **(A)** Moderate individual EPSGs (only 6 or more coincident inputs exceed threshold for spike from the resting state): 5nS (S), 2.5nS (D), 3.5nS (C). **(B)** Strong individual EPSGs (only 4 or more coincident inputs exceed threshold for spike from the resting state): 7.5 nS (S), 3.75nS (D), 5.25 (C). Computations were performed with 1000 cycles.

We adopt a stochastic input framework to characterize the models' coincidence detection properties for cyclic drive. Each cycle has a fixed number of inputs; they are more or less time coherent for higher or smaller values of *b*, respectively, according to a von Mises distribution (Figure 6 and Methods). The probability of firing in a cycle varies with the input frequency and temporal coherence of inputs (Figure 7). The V-shape of these parameter regimes for firing is not unexpected. They resemble the diagrams for phase-locking of the models for periodic inputs, as for full or partially rectified sinusoidal current injection (Figure S1B and Gai et al., 2009, 2010; Meng et al., 2012). However, for deterministic periodic input the transition in input parameter values from 1:1 entrainment to low firing rate is sharp at low frequencies, here it is gradual because the composite input per cycle involves stochastically-timed unitary events. The curved nature of the high and low frequency legs of the V reflect the threshold of these models for an input's rising slope together with the feature that temporal coherence here is with respect to a cycle's phase not absolute time. Say, the need for higher *b* values to ensure firing for lower frequency inputs accounts for the need to have fast rising input. We also see here that the V's apex, around 200–300 Hz, is similar to that of S and C for sinusoidal input, suggestive of a resonant like property of these models (Remme et al., 2014). These models do not fire for our EPSG volleys for cyclic frequency above 400 Hz, regardless of the input temporal coherence *b*. Temporal summation of the inputs for τ_*s*_ = 0.3 ms leads to enough baseline activation of negative feedback to preclude spiking. This upper limit of frequency is larger if unitary inputs are larger (compare Figures 7A,B). Later, we will see that inhibition may restore the excitability properties in these situations, especially for the D model. Note that for the sinusoidal input of Figure S1B we allowed amplitude to be a stimulus parameter and therefore entrainment could be to very high frequencies.

For both moderate and strong inputs, (Figures 7A,B, respectively), the D model is less selective to time coincident inputs. The D model can spike with higher probability for weak temporal coherence than the S and C models; D has a lower threshold for input slope that causes firing (see Figure S1B). The D model's lower slope threshold is further seen in D's tolerance for low frequency inputs. The more stringent requirement of S and C for steeper input slope is also seen in the spike triggered averages (see Figure S4). For weakly coincident inputs (small *b*), only a few realizations lead to composite EPSGs that are steep enough to cause firing, hence the low firing probability for S and C models. This observation suggests that models with dynamic *I*_KLT_ are more sensitive to inputs arriving together while the D model, with frozen conductance for *I*_KLT_ is far less selective about how these inputs are distributed along a cycle.

The firing probability increases for each of the models if the unitary input strength increases (compare Figure 7A,B). Coincidence sensitivity persists with incresing input strength although the minimum degree of coincidence needed for firing decreases (leftward shift of colored region). The stronger unitary inputs lead to larger increments in rising slope per event. It is easier to generate with fewer inputs a fast enough rising composite input to cause a spike. The D model here as well fires less selectively than S and C; at 50% rate for very weak coincidence (*b* = 2, with VS~0.7) over a substantial input frequency range. This feature of the D model is likely reflecting the passive and slower time course of membrane potential decay after a subthreshold EPSP peak, in contrast to the active decay from *I*_KLT_ in the S and C models (Jercog et al., 2010; Khurana et al., 2011; Mathews et al., 2010). This allows more time for a subsequent EPSG to give another sharp rise to the input. The D model, although legitimately phasic by our definition, behaves somewhat like an integrator for summation of fast unitary inputs.

It is rather striking to notice that, for a fixed input frequency (say, 250 Hz in Figure 7A), the dependence of firing probability on *b* for the S and C models is considerably more gradual than for the D model. For *b* = 8 less than 50% of inputs rise fast enough to cause S to fire; for *b* = 20 about 70% are fast enough (see Figure 6B). For very large *b* (~ 35) the firing probability becomes close to one. Only when the event times are very tightly timed will the input's rise be treated as fast enough for S. For lower values of *b* the fraction of realizations (cycles) that have events so tightly timed is less and this dependence on *b* is gradual for S and C. The range of *b* values for which the firing rate rises from 0 to 1 can be considered as the dynamic range for coincidence detection. Although this dynamic range is wide for S and C the slope threshold is high and demands for detecting coincidence in a cycle's composite input are high. The S and C models with a large dynamic range have a firing probability that encodes the coincidence. The D model is less discriminatory. It will fire at high probability for far weaker coincidence and its firing probability is not well graded.

**Figure 8:**
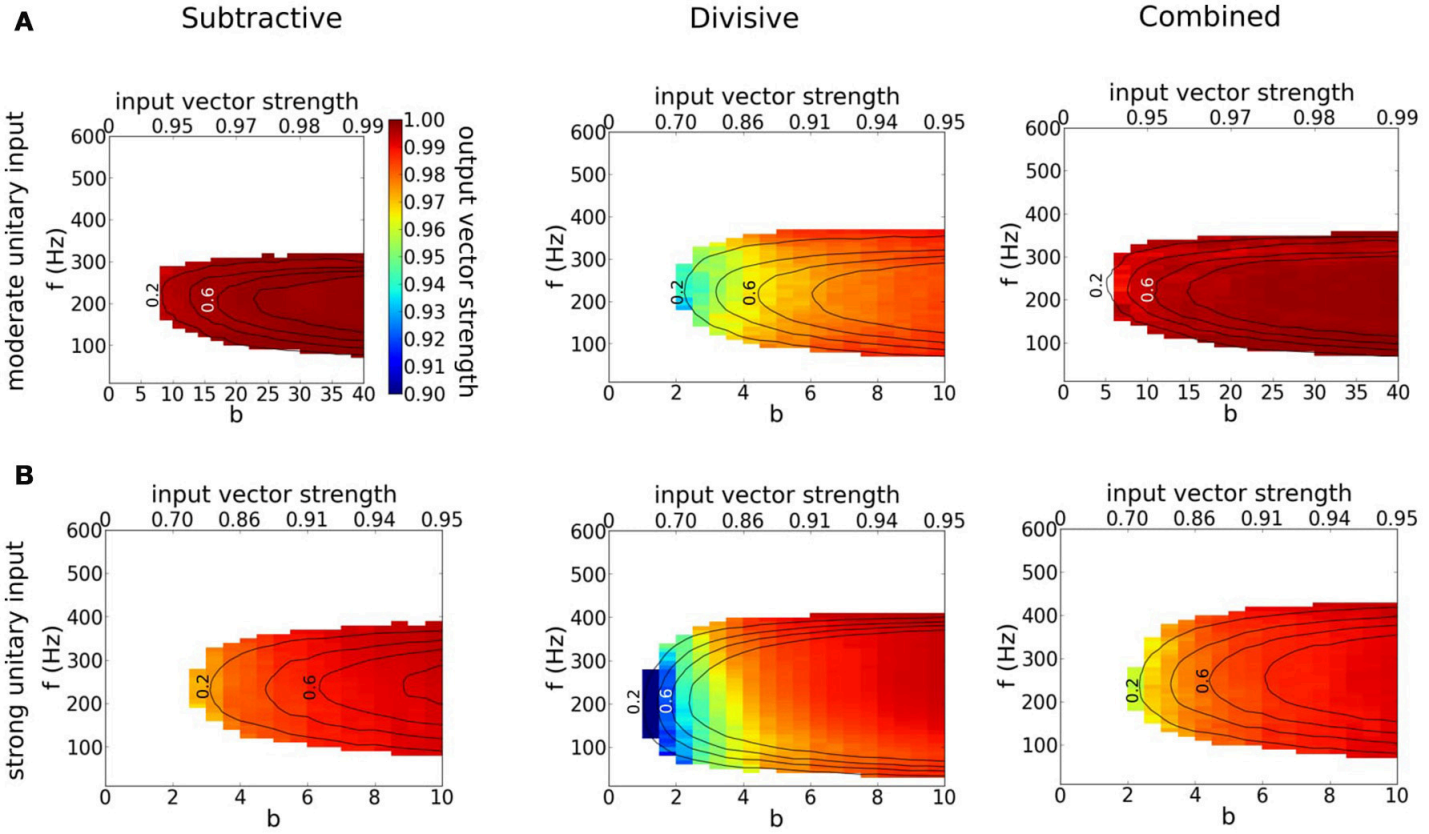
**Precision of phase-locking to a periodic train of multi-synaptic inputs with varying temporal coherence and frequency**. **(A,B)** Output vector strength for simulation runs in Figure 7 for the S (left), D (middle) and C (right) models as a function of temporal coherence in the arrival of inputs (*x*-axis; bottom axis shows temporal coherence parameter *b* in von Mises distribution, while top axis shows its corresponding input vector strength) and input frequency *f* (*y*-axis). **(A)** Moderate individual EPSGs. **(B)** Strong individual EPSGs. Contours correspond to output firing rate probability per cycle of 0.2, 0.4, 0.6, and 0.8 (spikes/cycle).

**Figure 9:**
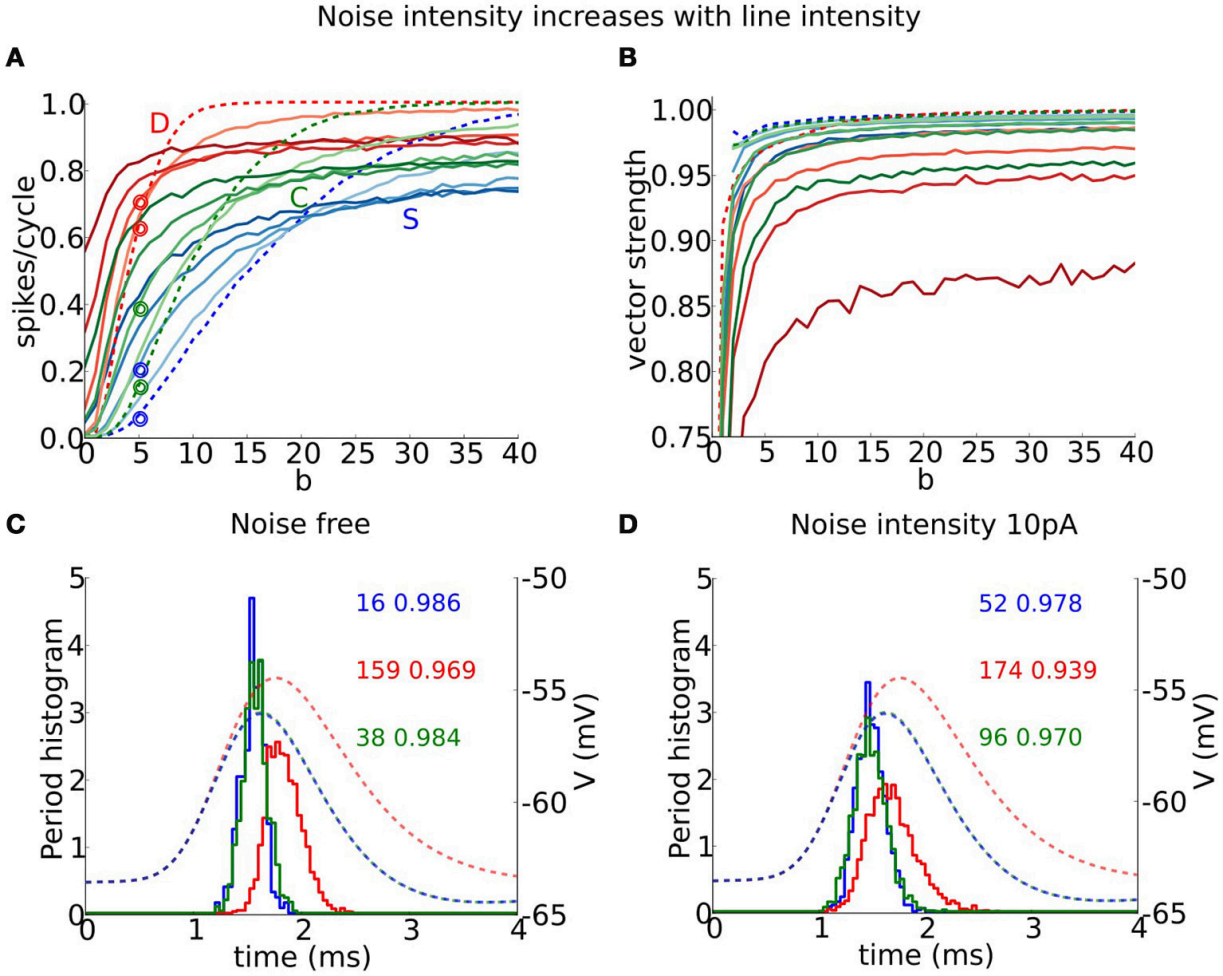
**Entrainment and vector strength properties for a periodic train of multi-synaptic inputs in the presence of background (white) noise**. **(A)** Output firing rate (spikes/cycle) and **(B)** vector strength for S (blue), D (red), and C (green) models for a periodic input of *f* = 250 Hz and low unitary vector strength (6 coincident inputs exceed threshold for spike from the resting state, as in Figures 7A, 8A) as a function of input temporal coherence *b* for different noise intensities: dashed line corresponds to the noise free system and color intensity indicates noise intensity ranging from 5 to 20 in steps of 5. **(C,D)** Period histograms (solid curves) for S (blue), D (red), and C (green) models for responses to inputs indicated with empty circles in **(A)**. **(C)** Corresponds to the noise free system and **(D)** to noise strength 10 pA. We show EPSPs (dashed curves, notice that blue and green lie on top of each other) for the average composite EPSGs obtained from the summation of individual mEPSG with *g*_*max*_ = 1 (take note that this is not the maximal amplitude used in simulations). Numbers indicate firing rate (left) and vector strength (right) for the different models.

Each model phase-locks with high precision to the cyclic stimulus, as seen by the high vector strength, VS is above 0.9 in most of the area where responses exceed 0.1 spikes/cycles (Figure 8, non-white region). Interestingly, the output VS is substantially higher than the input VS (Figure 8, top horizontal axis), evidencing that these coincidence detectors can enhance the precision of entrainment. Although the three models seem quite similar in terms of VS, they differ significantly in terms of histogram shape (Figure 9C). Thus, models with dynamic *I*_KLT_, when they respond, they do so at a higher precision than the D model. This fact is especially noticeable at low input strength (see Figure 8A).

#### 3.6.2. Coincidence detection and precision of entrainment in the presence of white noise

Our coincidence detector models show varied responses in the presence of background (white) noise to inputs like those of Figure 7. For conditions of low firing probability (low *b* and low to moderate input frequency) the noise can boost subthreshold inputs and induce firing, thereby increasing detectability. However, for conditions of high (noise free) firing probability, noise reduces firing probability and some suprathreshold inputs go undetected—reliability is decreased. We found that precision is reduced by noise for all three models, but especially for D. We illustrate these effects for input at frequency 250 Hz with low unitary input strength (as in Figure 7A). For a given noise level, firing probability increases with *b*, initially, above the noise free probability but ultimately below (Figure 9A). As noise strength increases, the three models show an increased firing rate, for low to moderate *b*-values. The S and C models retain some gradation of firing rate with degree of coincidence, *b*, but the D model has a much sharper rise to saturation, thus compromising the rate encoding of coincident inputs. Moreover, the minimum value of *b* for spiking is substantially diminished in the D model; it fires spontaneously at high noise levels. The precision (measured as VS) decreases with noise in all models but far more for the D model than for S or C (Figure 9B).

The S and C models entrain with different phases than does D although noise induces more firing during the rising slope of the mean EPSP for each model in the low to moderate firing rate regime. Consider the case of 250 Hz input, as in Figures 9A,B. In the noise free case the firing times for the three models occur mostly at the peak of the EPSP for the average synaptic conductance (Figure 9C). With noise (10 pA here), the firing time distribution spreads more toward earlier firing corresponding to the rising phase of the EPSP, and this effect is more noticeable in the D model (Figure 9D). Most of the added spikes thus appear to reflect slope sensitivity, enhanced by the noise. This effect is reminiscent of earlier findings (Gai et al., 2009, 2010) in which firing to slow modulation could be induced on the rising phase when no firing occurred in the absence of noise, a feature referred to as slope-based stochastic resonance. Here, the spread of firing times carries the consequence of degraded precision, a trade-off for enhanced slope sensitivity. The D model is more vulnerable to this phenomenon due to its input resistance. We see here that small differences in noise (and thus input vector strength) translate into significant effects on the period histogram's shape, and on the firing rate for S and C, 2−3 times increased.

#### 3.6.3. The timing of brief inhibition can counter or promote firing

Inhibition can have contrasting effects, negative or positive, on firing probability. We showed such contrasts for steady inhibition (*g*_inh_) and Poisson distributed excitatory synaptic input events (Figure 5). For a substantial range of EPSG input rates, increasing *g*_inh_ leads to decreased firing probability – an expected outcome. However, for high EPSG input rates that lead to substantial reduction in excitability (the non-monotonic input-output feature of type III excitability), we found that *g*_inh_ can promote firing for the D model by decreasing membrane potential and thereby partially restoring excitability. Here, for repetitive excitatory input we show that brief inhibition may increase or decrease firing probability depending on the timing of inhibition and, further, that the promoting effect may be enhanced if inhibition is more sharply timed (higher *b*-value).

The familiar phenomenon of post-inhibitory rebound relates to the hyperexcitability following release from a sufficient duration of hyperpolarization that can cause a spontaneous spike or induce one from a subthreshold depolarizing input. Perhaps more surprising is that a brief, well-timed, hyperpolarizing inhibition can have a similar effect, leading to a spike for an otherwise subthreshold EPSG. This phenomenon, referred to as Post-Inhibitory Facilitation (PIF), has been demonstrated for the Hodgkin-Huxley model (Dodla and Rinzel, 2006) (Type II excitability) as well as for auditory brain stem neurons and the RM03 model (Dodla et al., 2006) (Type III excitability). Here, we characterize and compare the timing window, intensity range and VS range for reduction and facilitation of firing probability for the S, D, and C models. The excitatory stimulus is a repetitive von Mises distributed event train as we used for characterizing features of entrainment (Figures 6–8). Each cycle includes an inhibitory input (von Mises) with a specified phase difference with respect to excitatory input (they are coincident at phase 0, see Section Methods). We will consider two different input frequencies: a low frequency (150 Hz) for which the excitatory input in one cycle has little effect on the next cycle; and a high frequency (380 Hz), for which temporal summation of excitatory inputs activates subthreshold negative feedback thereby affecting subsequent spike generation. For comparing the influence of inhibition features we choose to define our control conditions so that each model has an approximately identical firing rate. We describe first the results for low frequency input with control conditions: for each model, set an EPSG value so that 6 simultaneous inputs elicit a spike and set the *b*_*e*_ value to have a firing rate of about 50 Hz.

**Figure 10:**
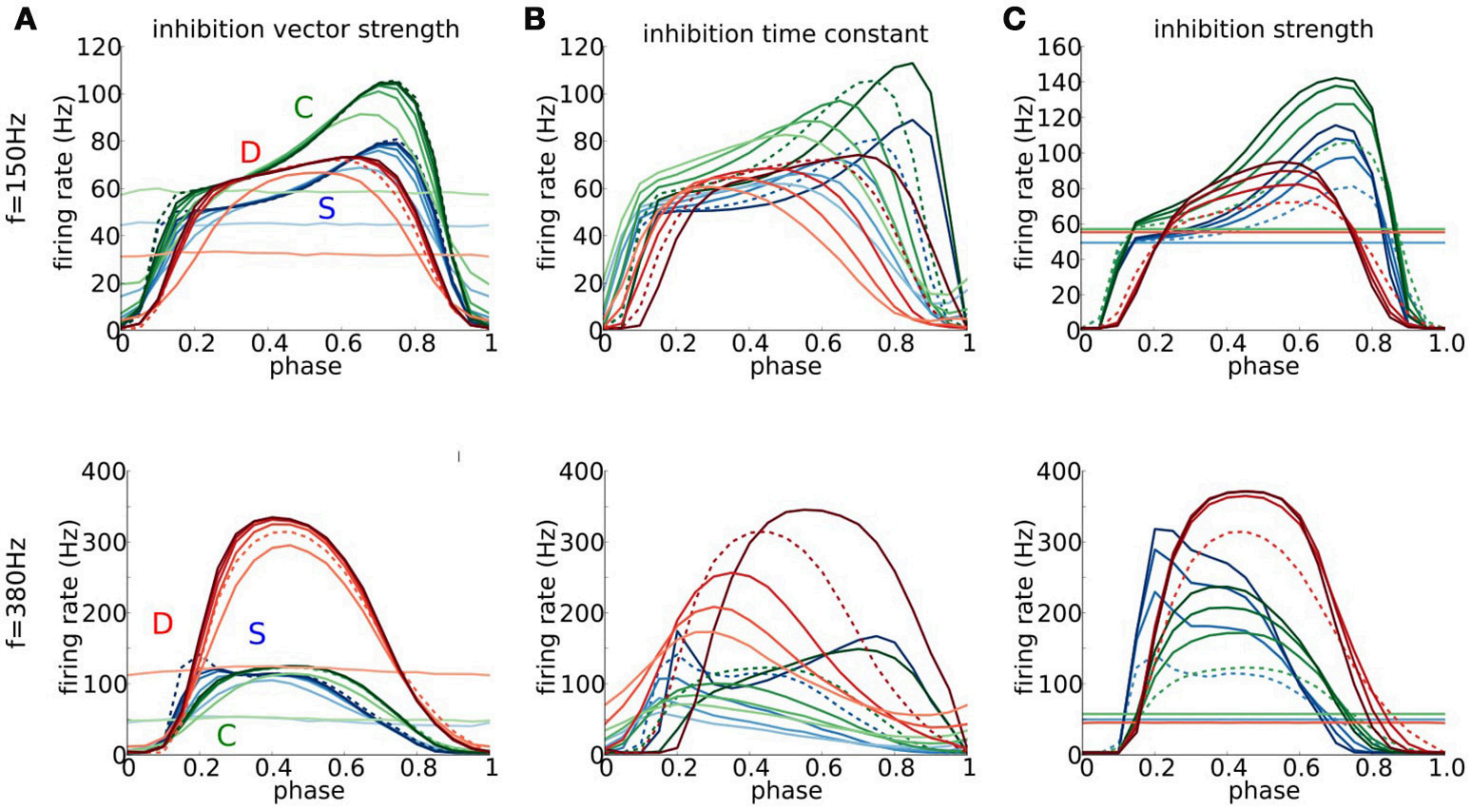
**The role of timed inhibition in signal detection at low and high frequencies**. **(A–C)** Output firing rate for periodic trains of combined excitatory and inhibitory multi-synaptic inputs for the D (red), S (blue), and C (green) models, as a function of phase of inhibition with respect to excitation for *f* = 150 Hz (top) and *f* = 380 Hz (bottom). Parameters for the excitatory input are fixed so that the output firing rate in the absence of inhibition is about 50 Hz (for *f* = 150 Hz, *b*_*e*_ = 15 (S) *b*_*e*_ = 4 (D), *b*_*e*_ = 12 (C), and for *f* = 380 Hz, *b*_*e*_ = 15 (S) *b*_*e*_ = 3 (D), *b*_*e*_ = 4 (C)). Maximal conductance for mEPSGs *g*_*max,e*_ at *f* = 150*Hz* is chosen so that six coincident inputs exceed threshold and at *f* = 380*Hz* so that four coincident inputs exceed threshold. Parameters modeling the inhibitory multi-synaptic input are varied. **(A)** We vary the temporal coherence of the inhibitory inputs, ranging from *b*_*i*_ = 0 (light colors) to *b*_*i*_ = 10 (dark colors) in intervals of 2; here τ_*i*_ = 0.3 ms and *g*_*max,i*_ = 2.5 nS. **(B)** We vary the time scale of inhibitory inputs τ_*i*_, ranging from τ_*i*_ = 0.1 ms (fast, dark colors) to τ_*i*_ = 0.9 ms (slow, light colors) in intervals of 0.2 ms; here *b*_*i*_ = *b*_*e*_ and *g*_*max,i*_ is scaled so that the total area under the α-function modeling IPSGs remains constant and takes the same value as for τ_*i*_ = 0.3 ms. **(C)** We vary the inhibiton strength *g*_*max,i*_, ranging from *g*_*max,i*_ = 0 nS (light colors) to *g*_*max,i*_ = 10 nS (dark colors) in intervals of 2.5 nS; here *b*_*i*_ = *b*_*e*_ and τ_*i*_ = 0.3 ms are fixed. Dashed curves identify the curves in **(A–C)** that share the same parameters for inhibition.

The dependence of firing probability on the phase of inhibition shows a common feature nearly throughout our parameter variation set: firing probability is reduced nearly to zero when inhibition is timed close to excitation. This reduction effect can be very localized in time but for some other phases firing probability shows prominent increases. For time focused inhibition (*b*_*i*_ > 6, say) the firing probability increases by a factor of 1.5–2 for inhibition that just precedes excitation by 30–40% i.e., ≈2.5 ms (Figure 10A). This enhancement is more sharply tuned in phase for S and C than for D. The weaker phase dependence for D is consistent with the slower and monotonic decay of an IPSP in the case of D. The IPSP decay for S and C exhibits resonant like behavior with an overshoot and therefore a greater phase preference (see Figure S5). The phase ranges where the firing ratio is near one correspond to inhibition that arrives just after a spike in the previous cycle and too far ahead of the next excitatory event to have any effect on firing probability. Finally, if inhibition is not focused in time but rather smeared (low value of *b*_*i*_) the firing probability is close to or less than the control value and without phase preference.

The time course of inhibitory transients significantly affects the PIF mechanism. If *g*_inh_(*t*) is too fast or too slow, relative to the intrinsic timescales of *V* and the subthreshold negative feedback, PIF is precluded as shown in Dodla et al. (2006) for the *I*_KLT_ mechanism. For our models and with repetitive excitatory input, as τ_*i*_ decreases and becomes small enough, the briefer *g*_inh_ leads to a sharpening of the phase preference (Figure 10B) and stronger enhancement in firing probability. In contrast, as τ_*i*_ increases the phase preference tuning broadens and enhancement reduces giving way to a broadening of the late phase range in which firing probability is reduced below the control level (~50 Hz). This reduction is especially true for the D model; for τ_*i*_ = 0.9 ms the firing probability is below control values for approximately 80% of the cycle. The slower decay of an IPSP for D than for S and C (again, Figure S5) is exaggerated by larger τ_*i*_.

Stronger inhibition that is focused and brief can enhance the PIF effect (Figure 10C). The firing probability increases and the phase preference sharpens for phases just before the excitation event (approximately 1.7 ms in advance). The behaviors resemble those for increasing the vector strength of inhibition (cf, Figure 10A).

Notice that the C model exhibits in Figure 10 (top) more enhancement of firing probability by brief inhibition overall than D or S, in contrast to our comparisons of other response features (say, Figures 2, 4, 7) where C appeared intermediate in degree. The difference is reflecting the fact that the C model includes both mechanisms of subthreshold negative feedback. Hence, transient inhibition is capable of inducing extra hyperexcitability.

The behaviors for high frequency (380 Hz) excitatory input differ somewhat, but not surprisingly, from those for low frequency input (Figure 10, lower panels; see caption for control conditions). Overall, the D model shows the greatest enhancement in firing probability with transient inhibition. This effect is understandable since hyperpolarization from transient inhibition must overcome for S and C two factors: the temporal summated excitatory conductance between input events (the only hurdle for D) and the accumulated *g*_KLT_. The D model shows less phase preference for PIF. For S and C the preferred phases are shifted earlier. These shifts correspond to 1.6−2.0 ms before the next input event (comparable in absolute time to that for the low frequency input case), a time scale comparable to τ_*w*_ and τ_*h*_. Interestingly, here for high frequency input the C model shows a much weaker PIF effect than S, although having a slightly larger effect than S for low frequency input.

**Figure 11:**
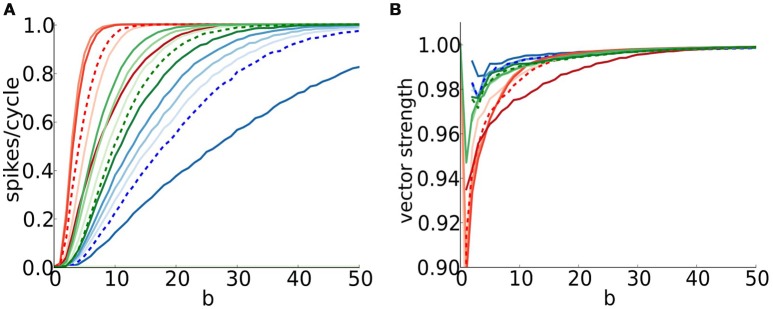
**The timing of inhibition can affect coincidence detection and vector strength**. **(A)** Firing probability (spikes/cycle) and **(B)** vector strength for S (blue), D (red), and C (green) models, input frequency *f* = 250*Hz* and low input strength (six coincident inputs exceed threshold for a spike) as a function of input temporal coherence *b* (*x*-axis) and phase difference between inhibition and excitation indicated with color intensity of curves (0.2 = light and 0.8 = dark in steps of 0.2). Recall that for values of phase difference close to 0, inhibition just follows excitation, while for values close to 1, inhibition just precedes excitation. We include the results for no inhibition (dashed curves) for comparison.

While timed inhibition can boost firing probability for peri-threshold random synaptic events it can also enhance coincidence detection and precision. We assessed in Figure 7 coincidence detection quality by showing the dependence on *b* (the degree of temporal coherence in the input) for repetitive excitatory input; a horizontal slice in the heat map shows, for a given frequency, the increase of firing probability vs. *b* (Figure 11A, dashed curves for 250 Hz input and low strength, as in Figure 7A). The effect of brief inhibition on this sensitivity to coincidence depends on the phase of inhibition (indicated with color intensity). In this view we recover the impression that the C model responds to coincidence with sensitivity intermediate to that of S and D; the firing probability and the “dynamic range” (interval of *b*-values for gradation of firing probability) are intermediate. The S model, with its subtractive mechanism, has the largest dynamic range while the D model is coincidence-sensitive over only a narrow range before saturation with firing probability exceeding 0.9 for *b* > 5 or so. For each model we see that even when inhibition occurs close to excitation (dark curves) the low firing probability can be increased with coincidence. These results suggest that timed inhibition may modulate the rate encoding and dynamic range for coincidence detection of phasic cells, creating windows over different *b* ranges.

The temporal precision (vector strength) of the models' responses increases with temporal coherence (Figure 11B). Comparison between the models shows C as intermediate and D with least precision. For timed inhibition, a high VS can always be achieved for strongly coincident inputs. In contrast, the VS is limited in the presence of noise even for very high *b*-values (compare Figure 9B and Figure 11B).

## 4. Discussion

Phasic neurons do not respond repetitively to steady inputs. The primary mechanism for phasicness is a dynamic negative feedback mechanism that activates for subthreshold values of voltage, preventing the generation of a spike. Here we explored two mechanisms of different nature: a potassium current (outward current) that activates subthreshold and opposes depolarization (subtractive mechanism) and a transient sodium current (inward current) that inactivates subtreshold and can overwhelm activation gating of the regenerative current (divisive mechanism). We considered two reduced models that isolated these mechanisms, one that has only subtractive mechanism (S model), and one that has only divisive mechanism (D model); and a third model that combined both (C model), and compared their performance under different stimuli.

We found that each mechanism, divisive and subtractive, alone can support type III excitability. We do not advocate choosing one, say subtractive, as the default without consideration of the other. By extension, one should not assume that if an *I*_KLT_ blocker is applied (or *g*_KLT_ is replaced with a frozen *g*_KLT_, by dynamic clamp) and type III persists that *I*_KLT_ is not playing a role in Type III. The effect could have unmasked a D-mechanism, meriting further study.

Firing in type III excitability requires that the voltage outraces the dynamic negative feedback before this feedback can activate significantly and disallow a spike. This speed requirement on voltage rise means that these neurons and neuron models can act as input slope detectors. We found that our model with a subtractive mechanism requires a faster input rise than models without it (see ramps and sinusoids in Figure S1). Indeed, the (subtractive) low-threshold activated potassium current directly competes with a rising voltage by providing high conductance, that shunts EPSCs as well as noise fluctuations. The divisive mechanism of Na^+^ inactivation does not interfere with the rising voltage but rather hinders the regenerative power of the sodium current. For this reason, we assert that cells equipped with *I*_KLT_ are better-suited for temporal processing like phase-locking and coincidence detection and with extraordinary temporal precision than neurons with only subthreshold Na^+^ inactivation (see Figures 7, 8).

However, neurons without sodium inactivation, need to strongly reduce the maximum Na^+^ conductance in order to preserve type III excitability, thus compromising excitability and ultimately signal detection (see Figure 3). Neurons equipped with both mechanisms, show the best combination of coincidence detection and signal detection phasic properties in addition to being the more robust to variations in channel densities. Indeed, the subtractive mechanism, when accompanied by a sodium current that inactivates (even if not subthreshold), increases its excitability while maintaining phasic properties. The divisive mechanism, if combined with a subthreshold activated potassium *I*_KLT_, becomes more selective to coincident inputs.

We provided new clues to assist in hypothesizing about mechanism(s) that may underlie phasic behavior in a given system. For instance, one can expect very differently shaped input-output relations (Figure 4) depending on whether S or D is dominant in determining Type III behavior; the relations for D are rather symmetric and not so for S and, for S, the asymmetry interestingly switches if firing probability is plotted vs. input strength or plotted vs. 〈*V*〉. Moreover, the effect of increased inhibition onto these curves shows different trends: decreasing the firing rate monotonically in the S model while affecting firing rate non-monotonically in the D model (Figure 5).

Given a particular system, one's approach for investigating and rationalizing the mechanistic basis should consider the context, the firing regimes. For instance, one could imagine a neuron with phasic spike capability at hyperpolarized levels, with an *I*_Na_ that differs from the *I*_Na_ that supports spike capability at depolarized levels. In this case, we might predict in favor of a D mechanism and against S as the default. Indeed, S would involve a substantial resting conductance that would require exceptionally strong excitatory input to elicit spikes with depolarization.

### 4.1. Type III excitability, differentiators *par excellence*

Neurons that mainly fire due to rapid depolarization to threshold caused by time coincident inputs (like the models considered herein) are often called differentiatiors (Izhikevich, 2007; Prescott et al., 2008a; Lundstrom et al., 2009; Ratte et al., 2014). Although some neurons may display features of integrators and differentiators depending on the temporal pattern of the inputs (Gutkin et al., 2003; Rudolph and Destexhe, 2003), here we have focused on pure neural differentiatiors which, because of their intrinsic properties, are incapable of responding to temporally integrated, slowly varying input. This property is strongly linked to the concept of type III excitability. That is, the system approximately tracks a slowly varing input as a pseudo steady state -there is no bifurcation to oscillatory behavior with the input treated as a parameter. Input variability is the essential cause of spiking (Lundstrom et al., 2009, 2008). This excitability type complements the classical type I and type II excitability classes, where a stable fixed point destabilizes through a SNIC or Hopf bifurcation, respectively (Rinzel and Ermentrout, 1998; Borisyuk and Rinzel, 2005; Izhikevich, 2007; Prescott et al., 2008a; Meng et al., 2012; Rinzel and Huguet, 2013).

Neurons with type II excitability (emergence of repetitive firing via a Hopf bifurcation) are typically classified as resonators since the emergence of repetitive firing is via a Hopf bifurcation and thereby associated with subthreshold damped oscillations (some type III models can also show damped oscillations and thus resonator properties Prescott et al., 2008a,b; Mikiel-Hunter et al., 2016). However, such a system may display differentiator-like features when operating near but outside its repetitive firing regime (Prescott et al., 2008a; Ratte et al., 2014). The sensitivity to timing of multiple inputs, coincidence detection, is naturally inherited from the time windows of opportunity provided by subthreshold resonance. Some differentiator properties that we studied here are found exclusively in type III excitable systems. For instance, the fact that, in the presence of noise, pure differentiators have highly non-monotonic input-output curves and its firing rate is sensitive to variance and insensitive to mean input in the noise free case (see Figure 4). Moreover, in the present study, we added an extra ingredient: when the non-monotonic input-output curves are plotted as a function of the mean voltage (see Figures 4B, 5B), the firing rate decay occurs for subthreshold values of voltage (below ~−40 mV; when *m* is half activated), irrespective of the negative feedback mechanism considered. This property does not occur for type II neurons, for which the firing rate increases with mean input and the decay in firing rate may occur but at a much higher activated state.

### 4.2. Two mechanisms are better than one to provide robustness to neuron models

Previous studies on specialized coincidence detectors in the auditory brainstem (Manis and Marx, 1991; Rathouz and Trussell, 1998; Svirskis et al., 2002) have highlighted the relevance of *I*_KLT_ in conferring phasic properties. Our early finding that Na^+^ current in MSO neurons inactivated at low-voltages suggested that this feature contributes to enhancing coincidence detection (Svirskis et al., 2004). Here, we underscore that Na^+^ inactivation plays a strong role in guaranteering the functional properties of the neuron model in terms of both spike preservation and phasicness. Thus, if Na^+^ inactivation is disabled, as in the S model, sodium conductance must be tightly constrained in order to preserve the spike and avoid the “lockup” state (see Figure 3). We tried but were not able to counteract “lockup” and retain phasic behavior with an additional potassium current that activated at high voltage (see Appendix B in Supplementary Material). With the divisive mechanism alone, spiking is preserved if *h*_∞_ is left-shifted to lower values of voltage – *I*_Na_ must be strongly inactivated at rest – to retain type III excitability and avoid repetitive firing in response to steady current. However, having both types of negative feedback mechanisms in the neuron model (C model) provides a larger parameter space for conductances over which the system preserves phasicness, without compromising the spike or the excitability of the system. Thus, phasic models equipped with two mechanisms of different nature are more robust to changes in channel density (see Figure 3A).

In our analysis we considered extreme cases for our models. Indeed, it is not necessary that a C model, being phasic and performing properly, remain phasic when the subtractive mechanism is disabled. For example, the original RM03 model is phasic but behaves tonically when *I*_KLT_ conductance is frozen (Meng et al., 2012). Actually, there is a range of admissible positionings of Na^+^ inactivation for a C model that guarantee similar phasic features as our C model, even when the D model (frozen *w*) is not phasic. However, as *h*_∞_ is right-shifted, the phasic region for the C model is reduced and when the shift is too dramatric, it resembles our S model. In this sense, our S model is a limiting case of the C model. Thus, our parameter choice for models allow us to, on one hand, clearly label them as S or C and on the other hand, keep the parameter values as similar as possible for a fair comparison. See more details in Figure S6.

We have considered the idealized situation of a point neuron model, without regard for potential effects of differential spatial distributions of ion current mechanisms for phasic behavior. MSO neurons provide a case for such considerations. In MSO neurons somatically recorded spikes are typically quite weak *in vitro* (Scott et al., 2007) and distinguishing what might be spikes or synaptic transients is challenging from *in vivo* extracellular recordings. Spikes that are generated in the axon initial segment are presumably shunted by the considerable conductance of *I*_KLT_ and *I*_*h*_ in the soma-dendritic membrane (Khurana et al., 2011), even though Na^+^ channels (inactivated near rest) are present in the soma (Scott et al., 2010). A thorough modeling study of how MSO phasic firing may depend on both *g*_KLT_ and Na^+^ inactivation could include the spatial distribution of the various *V*-gated currents (Ko et al., 2016); the influence of spatial distribution should likewise be considered in other phasic firing neurons. MSO neurons are superb at computing time differences. In gerbil, they receive fast excitation and fast, soma-targeted, inhibition (Myoga et al., 2014). Perhaps the sodium current while inactivated at rest may be recruited (de-inactivated) by transient inhibition to respond selectively to well-timed inhibitory-then-excitatory event pairings as in post-inhibitory facilitation (Dodla et al., 2006).

Our findings can be extended to analyze the role of negative feedback processes in a wide range of biological rhythms. Indeed, the presence of two mechanisms of different nature opposing to depolarization is not exclusive of phasic systems. Thus, the original Hodgkin-Huxley model has also Na^+^ inactivation and K^+^ activation, which in this case, act mainly superthreshold. To counteract a strong non-inactivating inward current with a strong outward current constrains the allowable channel density. However, controlling the effect of autocatalysis by a divisive mechanism, makes the system less sensitive and more flexible to changes in channel density (Sengül et al., 2014). Spiking, crucial for neuronal communication and computation, needs to be preserved with respect to variations in channel density. A similar statement about “two better than one” can be applied to a network of excitatory and inhibitory neurons, say as in a firing rate framework like the Wilson-Cowan equations. In the classical formulation, inhibition acts as a subtractive mechanism to counter recurrent excitation (equivalent to sodium activation). Dynamic synaptic depression on the excitatory-excitatory interaction behaves divisively like sodium inactivation by directly controlling and reducing autocatalysis. Having both mechanisms enables a network model to behave phasically (Tabak et al., 2006, 2011).

In further regard to robustness and parameter choices in our study, we acknowledge some compromises. Since we needed to choose a low value of ḡ_Na_ for the S model, the excitability for this model was reduced. Thus, in order to keep a fair comparison with the other models, we also reduced ḡ_Na_ for D and C models with respect to the original RM03 model, although this was not necessary since D and C still are type III for a larger range of ḡ_Na_ (see Figure 3A). Lowering Na^+^ conductance reduces, of course, the excitability properties of the system, and leads to lower amplitude spikes. Lowering ḡ_Na_ was also suggested in Lundstrom et al. (2008) as a mechanism to turn an integrator into a differentiator.

### 4.3. The role of inhibition in restoring excitability and shaping coincidence detection properties

Transient inhibition can reduce or, surprisingly, enhance firing probability for peri-threshold random synaptic input events, depending on the timing and precision of the inhibition (Dodla et al., 2006). This property of post-inhibitory facilitation (Dodla et al., 2006) is a transient analog for post-inhibitory rebound after prolonged hyperpolarization. It can be expected in any system with a dynamic excitability-suppressing factor that is partially activated at rest and reducible by transient hyperpolarization or in spontaneously firing conditions for randomly arriving excitatory and inhibitory events (Dodla and Rinzel, 2006). As a model, we considered the response sensitivity for periodically delivered, compound excitatory input events (say multi-synaptic inputs) that are near to threshold but by chance are sometimes subthreshold/superthreshold and more or less time dispersed. Our models (S and C) with *I*_KLT_ express the behavior. Inhibition reduces the excitability-suppressing *I*_KLT_ current and although simultaneously positioning membrane potential further from threshold it opens a time window of enhanced excitability if inhibitory conductance decays fast enough (see Figures 5, 10). However, when excitability suppresion is primarily due to Na^+^ inactivation as in the D model, hyperpolarization from a transient inhibitory input can remove some Na^+^ inactivation and thereby effectively lower the spike threshold in spite of hyperpolarization. A normally subthreshold excitatory input may then trigger a spike even if inhibition has not completely decayed away (see Figures 5, 10). The C model should behave more as the S model or the D model depending on the contribution of each mechanism. Thus, the PIF phenomenon for the D model is less sensitive to the arrival time of the inhibitory inputs, compared to S and C, and this is especially noticeable at high frequencies.

While timed inhibition can facilitate signal detection, it can also enhance coincidence detection and precision. Several studies have shown that timed inhibition close to excitation, either preceeding or following excitation, can improve temporal precision of neurons (not only neurons with type III) by narrowing the window for coincidence detection, sometimes at the price of reducing the firing rate (Brand et al., 2002; Grothe, 2003; Ingham and McAlpine, 2005; Kuenzel et al., 2011). Indeed, we observed that timed inhibition close to excitation can deselect some spikes that do not correspond to coincident inputs and improve precision, especially for the S and C models, but also modulate the rate encoding and dynamic range of temporal coherence for coincidence detection (interval of *b*-values for gradation of firing probability), creating windows over different *b* ranges (see Figure 11).

### 4.4. Generality of the model and the role of other currents

Many of our results are demonstrated for a specific conductance-based model (adapted from Rothman and Manis, 2003). However, the geometrical analysis and underlying mathematical structure of our reduced versions of the model suggest that qualitative aspects of phasicness will be found in a more general class of phasic models. Take for instance, Clay's model for a healthy squid giant axon (Clay et al., 2008), that was obtained from the original Hodgkin-Huxley (HH) model by steepening and left-shifting the activation of *I*_K_. The modified model can be seen as our C model, since HH has sodium inactivation. Another possibility is to turn the standard HH model into a type III excitable model by lowering the conductance of *I*_Na_ from 120 to 83 mS/cm2 (Lundstrom et al., 2009). In this case, the model can be seen as our D model but with a potassium current that activates superthreshold or close to threshold.

Indeed, we may have kept the high threshold potassium current *I*_KLT_ (present in the original RM03 model) in the D model. The role of this current is to contribute to the repolarization of the membrane potential once the spike has occurred – recall that in the D model the repolarization is only due to the leak current – but it has no effect on the spike generation, since this current is not active at subthreshold values of voltage. For this reason, the results described for the D model will also follow if *I*_KLT_ is added, since they mainly involve the subthreshold dynamics. Some differences will be observed if the activation of *I*_KLT_ is shifted to lower values, closer to threshold (around −40 mV for instance). In this case, *I*_KLT_ will behave as an *I*_KLT_ current, so the model will become a C model, with proper scaling of conductances.

Similarly, the *I*_KLT_ current can also be included in the C and S models. For the C model, the inclusion of *I*_KLT_ does not change the behavior of the model significantly (Meng et al., 2012). The S model is different since the inclusion of *I*_KLT_ might compensate the persistent *I*_Na_, and allow for a larger value of ḡ_Na_. Unfortunately, when *I*_KLT_ is incorporated in the S model, the system then switches to type II excitability (see Appendix B in Supplementary Material for a detailed discussion on this topic).

In many phasic models one can find a hyperpolarization-activated cation current *I*_*h*_ (Rothman and Manis, 2003; Khurana et al., 2011). The current *I*_*h*_ is mostly activated below *V*_*rest*_ with an activation time constant of 200−300 ms and deactivation by membrane depolarization on the order of tens of milliseconds. In our reduced models we have frozen *g*_*h*_ to its resting value, which is small. Allowing dynamic *I*_*h*_ may have an influence only on those results that involve dynamics below *V*_*rest*_. This will constraint the range of voltages for negative currents, because *I*_*h*_ prevents excessive hyperpolarization, in the same way as *I*_KLT_ prevents excessive depolarization. Moreover, when *I*_*h*_ is activated the input resistance is lower at values of voltage below *V*_*rest*_, thus the responsiveness to noise is smaller. The effects of a dynamic *I*_*h*_ are more noticeable in the C and S model than in the D model. Indeed, *g*_KLT_ is frozen at the resting value for the D model, therefore, when voltage hyperpolarizes, dynamic *I*_KLT_ deactivates for S and C, while for the D model it remains partially activated causing a similar effect on input resistance as if *I*_*h*_ were present: input resistance is smaller and there is less probability to fire due to noise effects. Thus, the presence of *I*_*h*_ (with dynamic rather than frozen-at-rest conductance) will have little effect on the essential results regarding coincidence detection properties. Even in the presence of inhibition, when *I*_*h*_ may reduce the IPSP size, the results will remain valid but for a stronger inhibitory input.

## 5. General conclusion

Subthreshold subtractive and divisive mechanisms each may contribute to, and together synergize to enhance, a cell's phasic properties. If they work alone, cell's performance may be compromised. Thus, the subtractive mechanism confers extraordinary coincidence detection properties to the cell, but, if alone (with non-inactivating sodium current), is operative within a restricted parameter range. The divisive mechanism guarantees robustness of phasic properties, without reducing a cell's excitability, although with somewhat less precision.

## Author contributions

Conceived the theoretical framework: GH, XM, and JR. Designed the models and performed numerical simulations and mathematical analysis: GH and XM. Wrote the paper: GH, XM, and JR. Edited the manuscript: GH, XM, and JR.

### Conflict of interest statement

The authors declare that the research was conducted in the absence of any commercial or financial relationships that could be construed as a potential conflict of interest.
