# Supplementary material for: Phasic Firing and Coincidence Detection by Subthreshold Negative Feedback: Divisive or Subtractive or, Better, Both
Source: Front Comput Neurosci. 2017 Feb 2;11:3. doi: 10.3389/fncom.2017.00003 (PMC5288357; doi:10.3389/fncom.2017.00003)
Supplement: Supplementary file 1 [file Presentation1.pdf]

Supplemental Figures

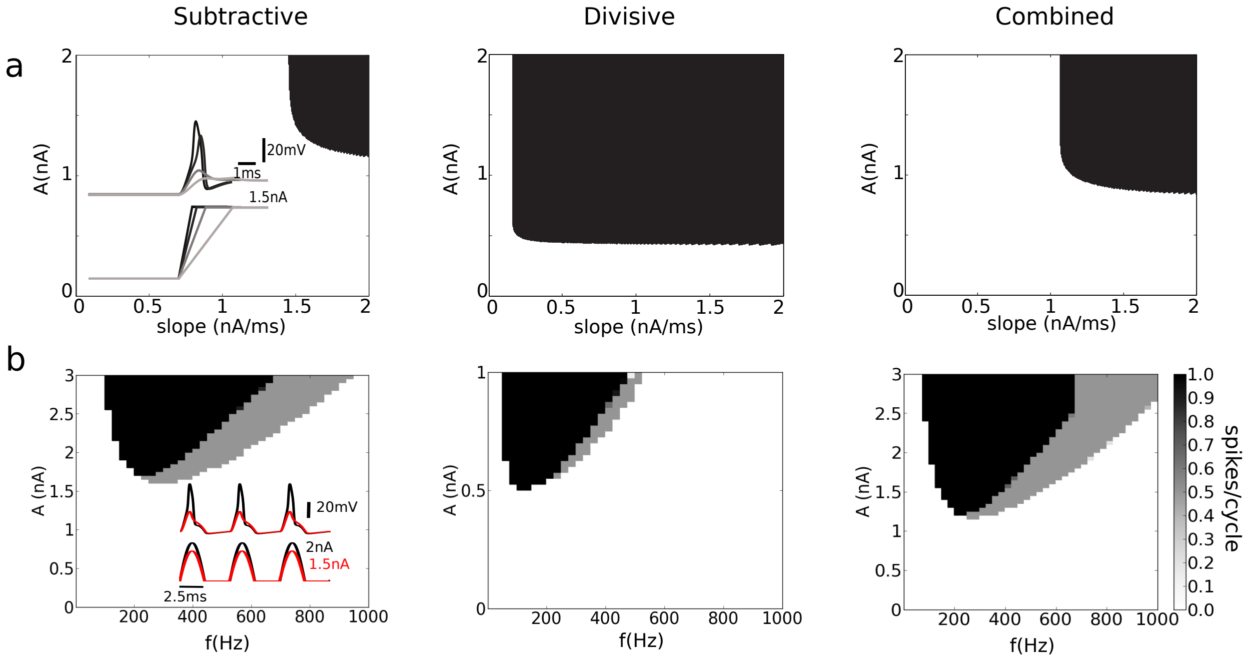

Figure S1: **Phasic neurons respond to fast-rising, but not to slow-rising, ramps.**(a) Number of spikes (black 1, white 0) in response to a ramp stimulus (inset) with varying slope (x-axis) and maximal amplitude (y-axis). A ramp current elicits an action potential only when its slope (dI/dt) exceeds some critical value, that is lower for D than for the other models. (b) Frequency-response maps of the models for a half-wave rectified sinusoidal input (equation 10) (inset) with varying frequency (x-axis) and amplitude (y-axis). Grey scale indicates number of spikes per cycle ranging between 0 and 1. Here, criterion for spike is V crossing -25 mV. The three models exhibit strong sensitivity to the increasing slope of current input rather than to the amplitude. With the same amplitude, the models do not respond to slow variations of the input (white, left of black region), but fire a single spike for high frequency variations in some range (black). The D model, however, can fire at lower frequencies than C and S and for lower values of the amplitude. For the D model we show only results for amplitude up to 1nA, since beyond this value the dynamics is totally dominated by the external input and the cell behaves passively. Notice that the best frequency (the one that requires the lowest amplitude for the input to cause firing) is around 200Hz for the three models.

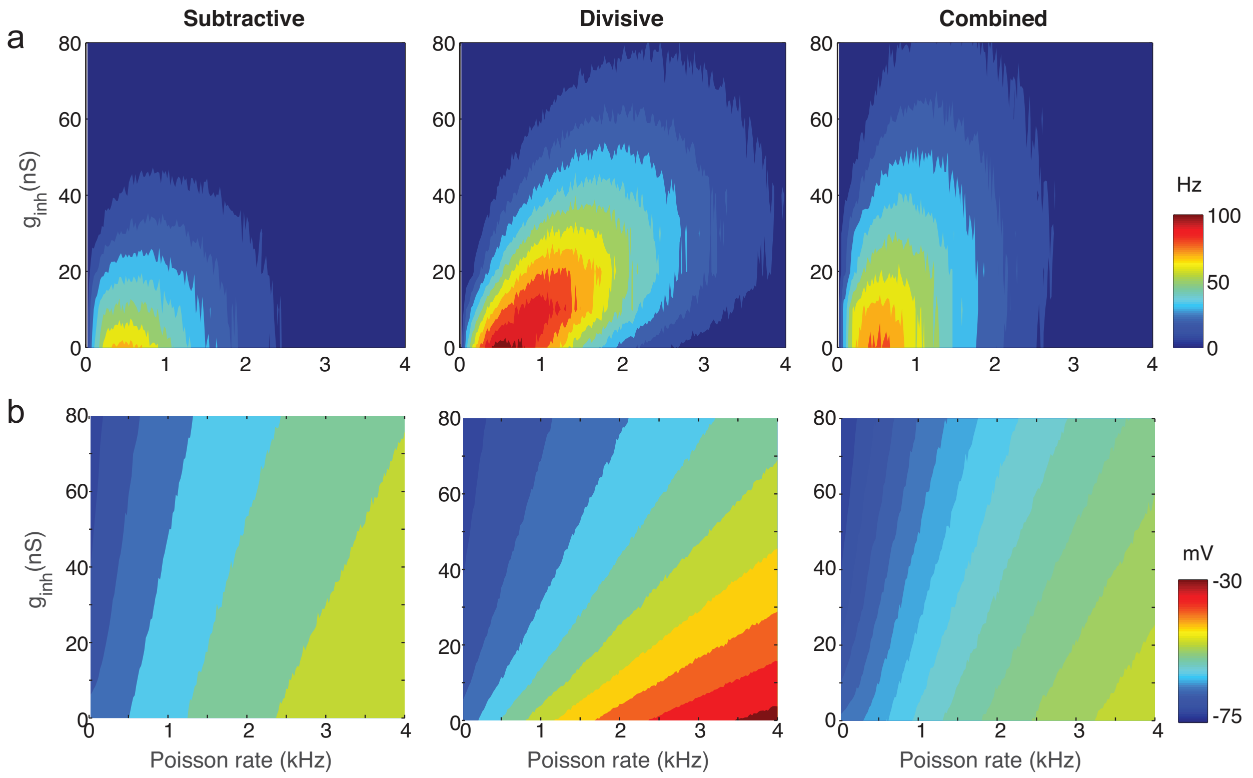

Figure S2: (continued on the following page) We show results as in Fig 5a but for a wider range of inhibition strength (from 0 to 80 nS). As in Fig 5, excitatory inputs consist of trains of synaptic conductance inputs (EPSGs) that are Poisson distributed in time with varying input rate (x-axis) and binomially-distributed in amplitude. The mean amplitude is different for each model and is chosen to produce an over-threshold EPSG probability of 50%, according to the threshold to elicit a spike from the resting state: 8 nS (S model); 3.86 nS (D model) and 5.7 nS (C model). Inhibition conductance is constant and varies from 0 to 80 nS (y-axis). (a) Contour plots of firing rate for S (left), D (middle) and C (right) models. Color indicates output firing rate (spikes/sec). Increasing inhibition strength decreases the firing rate for the S and C models monotonically, whereas for the D model the relationship between firing rate and inhibition is non-monotonic. Increasing inhibition reduces the firing rate for low EPSG rate (0-0.8 kHz), while for high EPSG rate (>0.8kHz) the firing rate first increases and then decreases. In Fig 5a we could only observe that inhibition increases the firing rate for high frequencies, here we can observe that when inhibition increases up to a large enough value then the output firing rate starts to decrease. We understand the counterintuitive effect of increased firing probability in D with g_{inh} for strong excitation as follows. The D model is without V-gated g_{KLT}. Thus, for high rate EPSGs there is substantial depolarization (Fig 5b) and inactivation of I_{Na}. However, g_{inh} can counteract the mean EPSG level and associated depolarization, $\langle V \rangle$, and therefore restore enough h, to make more sodium current available and increase the probability of spiking. For 2 kHz EPSG rate the firing rate is ∼20 Hz and with $g_{\mathrm{inh}} = 20$nS the firing rate increased to ∼50 Hz (green curve, Fig 5a), the associated drop in $\langle V \rangle$ was ∼10mV from a level of ∼-35mV (Fig 5b). Presumably the gaps in Poisson events (the same on average as without inhibition) and transient reduction of $\langle V \rangle$ increased the chances for spiking. For S and C, these levels of g_{inh} were inadequate to reduce $\langle V \rangle$ enough to increase spike probability (see supplemental Fig S2).

Figure S2: (continuation) The combined conductance, $\langle \text{EPSG} \rangle$ and g_{KLT}, overwhelms g_{inh} so there is only a modest decrease in $\langle V \rangle$; even though g_{KLT} is reduced with inhibition, the reduction in total conductance is apparently less than the increase that inhibition contributed so there is still strong conductance shunting. Thus, with the parameter settings for our S and C models the increase in firing probability for high EPSG rate was not observed. Perhaps for other parameter settings g_{inh} might enhance firing probability but we tried for decreased E_K (not shown) without finding the effect. (b) Contour plots of mean voltages ($\langle V \rangle$) corresponding to different Poisson EPSG rates and inhibition strength. $\langle V \rangle$ is computed by integrating sub-threshold voltages over time. It can be observed that unlike output firing rate, to keep $\langle V \rangle$ constant one must increase/decrease g_{inh} in the same direction as the EPSG input frequency. The decreased slope of constant $\langle V \rangle$ for the D model indicates that to maintain a balanced depolarization state of $\langle V \rangle$ the amount of effort of inhibition is smaller to overcome the increased excitation.

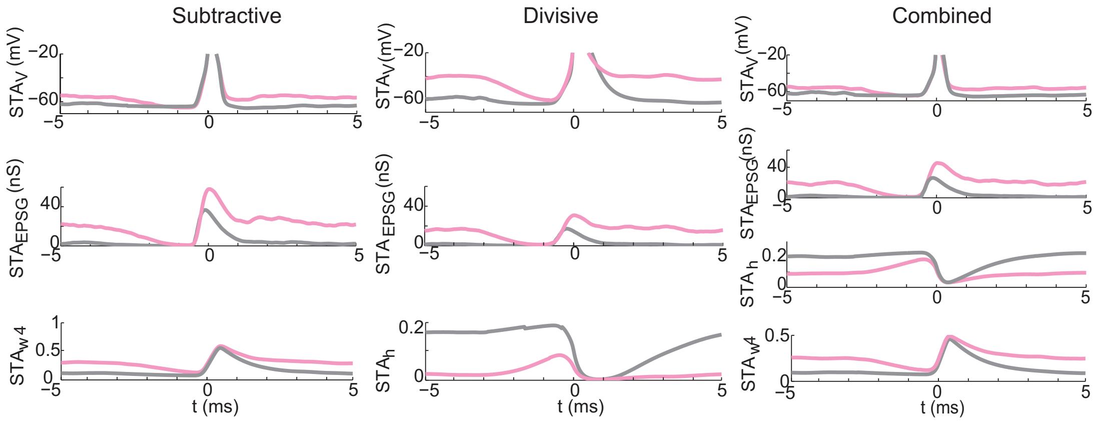

Figure S3: **Spike triggered averages (STA) for stochastic synaptic inputs**. The STAs of V, EPSG, w^4 (S and C models) and h (D and C models) are calculated by averaging those quantities 5 ms before and 5 ms after each spike. Two different input rates, one low (pink) and one high (grey) corresponding to gray and pink squares in Fig 5, respectively, are chosen for each model in order to generate approximately the same output rate (50 spikes/sec). The input Poisson rates are 140Hz and 1020 Hz (S), 140Hz and 1460 Hz (D) and 140Hz and 1280 Hz (C). For high EPSG rates (grey curves), spikes occur on average because there are gaps in the Poisson-timed EPSG arrivals, the summated conductance drops for about a ms and then, by chance, a large EPSG arrives or several EPSGs accumulate in a brief interval of time (see EPSG STAs). This reduction in the excitatory input causes a decrease in the mean voltage (see V STAs), removal of I_{KLT} and/or recruitment of I_{Na} (see w^4 and h STAs), facilitating the generation of spike when a subsequent large EPSG arrives. For low EPSG rates (pink curves), mean voltage remains close to the resting state (see V STAs), and then the arrival of a strong EPSG is enough to cause a spike.

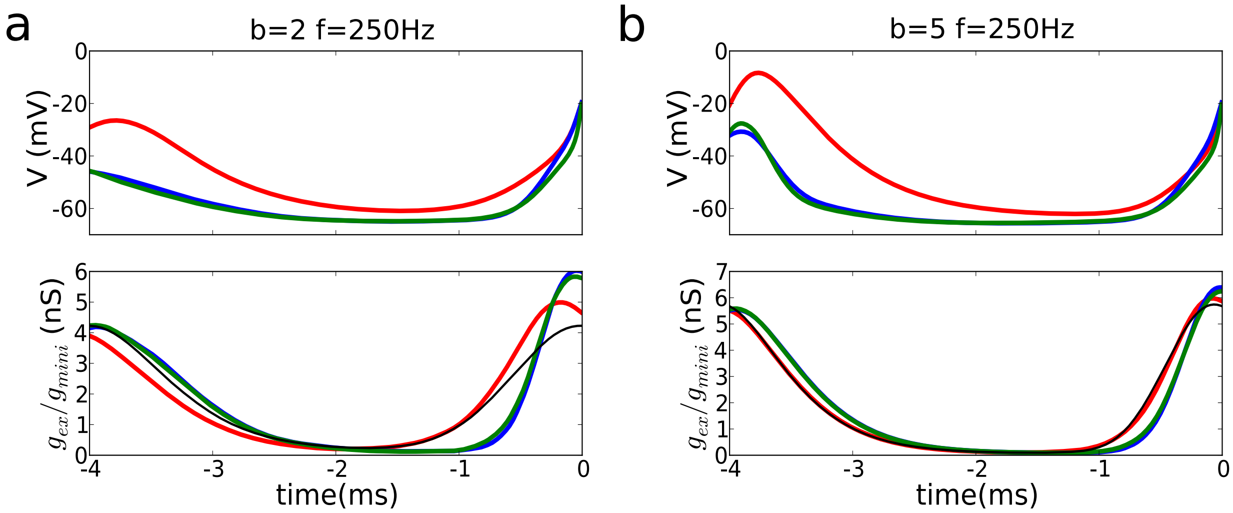

Figure S4: **Spike triggered averages for periodic multi-synaptic inputs**. Spike triggered averages for voltage (top) and composite EPSG normalized by the maximal size of the individual mini-EPSG (bottom) for S (blue), D (red) and C (green) models together with the averaged composite EPSGs (black curves) corresponding to input frequency $f = 250Hz$ and high input as in Fig 7b and for temporal coherence in the von Mises distribution $b = 2$ (a) and $b = 5$ (b). Recall that D model can spike with higher probability for weak coincidence than the S and C models. Indeed, we can see in the STAs that the D model has a lower slope threshold than S and C both at $b = 2$ and $b = 5$. For weakly coincident inputs (both $b = 2$ and $b = 5$), only a few realizations lead to composite EPSGs that are steep enough to cause firing (steeper than the average, compare green and blue curves with black one), hence the low firing probability for S and C models. While for the D model, for $b = 2$, it requires an input that it is steeper than the average, thus firing probability is less than 1 here, but for $b = 5$ the required slope for firing is already comparable to the mean synaptic input at this temporal coherence value, thus firing probability is 1 here (see Fig 7b). This shows that the D model is less selective to time coincident inputs.

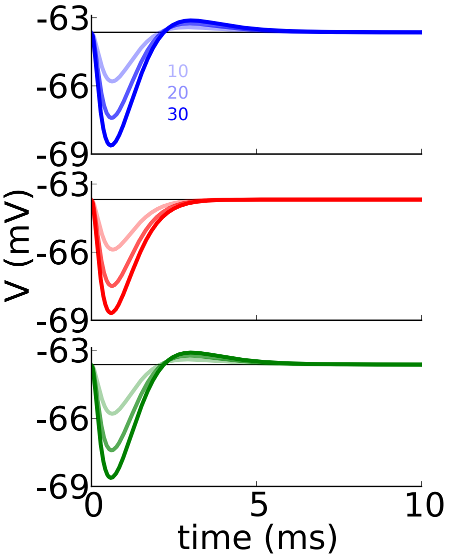

Figure S5: **Inhibitory Postsynaptic Potentials (IPSP) explain phase preference for timed inhibition.** IPSPs for the the S model (blue, top), D model (middle, red) and C model (bottom, green), in response to an inhibitory synaptic input modeled as an alpha funcion with time constant τ_i=0.3ms and varying maximal amplitude: 10nS (light curves), 20nS, 30 nS (dark curves). Horizontal black line corresponds to the voltage resting state and is included for reference. The IPSPs for the D model show a slower and monotonic decay, while for S and C models exhibit resonant like behavior with an overshoot. The S and C models take advantage of this resonant behavior and show a clear preference for the arrival time of inhibition. Thus, inhibitory inputs arriving about 2ms ahead of excitation have a stronger positive effect on the firing rate. On the other hand, the D model shows a weaker phase preference, with a larger range of phases for inhibition to have a positive effect on the firing rate (although weaker than S and C at low frequencies) and a wider peak locaded a few ms ahead of the peak for S and C due to the slower decay of its IPSPs (see Fig 10 top).

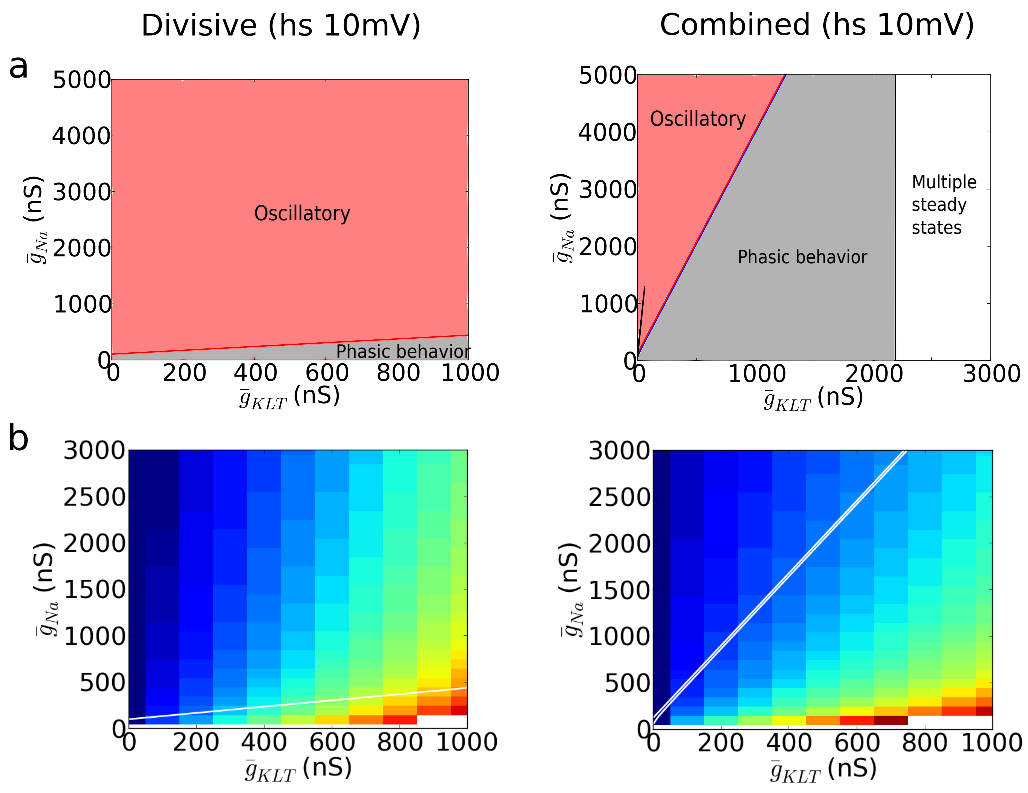

Figure S6: **Parameter range of phasicness is constrained for D and C models with right-shifted** h_∞. We show results as in Fig 3 for D and C models with h_∞ right-shifted by 10mV with respect to RM03 (16mV with respect to C and D models). (a) Different dynamic regimes are indicated with different colors. In the grey area the fixed point is stable for the biologically plausible range of voltages $V \in (-100, 40)$ (see Appendix A for more details) and no bifurcation occurs (type III excitability). In the red region, a Hopf bifurcation occurs (type II excitability). The white area has saddle-node bifurcations of the fixed points and multiple steady states are possible. (b) Minimal EPSG strength that produces a spike for an EPSG of the form $g_{ex}(t) = g_{max}(t/0.3)e^{1-t/0.3}$, assuming that the neuron is at its resting state. White area corresponds to no spikes. For the D model having I_{Na} inactivation right-shifted greatly reduces phasic properties. However, the C model is phasic over a robust parameter regime (only modestly compromised here by right-shifting h_∞), even when the D model (frozen w) is not phasic. When h_∞ is dramatically right shifted (not shown here) the phasic region for the C model is dramatically reduced and resembles the S model. In this sense, the S model is a limiting case of the C model (compare with Fig 3a left). Notice that S and C show similar dynamic behavior over similar input parameter ranges (Figs 4-5, Figs 8-9-10-11).
