# Supplementary material for: Phasic Firing and Coincidence Detection by Subthreshold Negative Feedback: Divisive or Subtractive or, Better, Both
Source: Front Comput Neurosci. 2017 Feb 2;11:3. doi: 10.3389/fncom.2017.00003 (PMC5288357; doi:10.3389/fncom.2017.00003)
Supplement: Supplementary file 2 [file Presentation2.pdf]

Appendix

A Mathematical analysis of 2D bifurcation diagrams

In this section we describe the mathematical analysis to compute the regions of stability described in Fig 3. Recall that we want to identify the regions of the parameter space $(\bar{g}_{\text{Na}}, \bar{g}_{\text{KLT}})$ for which the system has a unique fixed point which remains stable for the whole range of values of I that are biophysically plausible.

Let us consider a model in the general form:

$$\begin{aligned} C\frac{dV}{dt} &= -I_{ion}(V, x) + I, \\ \frac{dx}{dt} &= \frac{x_\infty(V) - x}{\tau_x(V)}, \end{aligned} \tag{18}$$

where $x = w$ in the S model, $x = h$ in the D model and $x = (w, h)$ in the C model.

We define the steady state current for the model as

$$I_{ss}(V) = I_{ion}(V, x_\infty(V)).$$

Notice that $I_{ss}(V)$ is a continuous function of V whose image spans the whole real space. If $I_{ss}(V)$ is a montone increasing function of V, then for a given value of I, there is a unique value of V that satisfies $I_{ss}(V) = I$ and the system (18) has a unique fixed point. Thus, if we impose the condition

$$\frac{dI_{ss}(V)}{dV} > 0 \quad \text{for all values of } V \in \mathbb{R},$$

we guarantee that the system does not undergo a saddle node bifurcation for any value of V, and the system has only one fixed point.

In our case, we look for values of $(\bar{g}_{\text{Na}}, \bar{g}_{\text{KLT}})$ for which there exist a value of $V \in (-100, 40)$ –the biologically plausible range– such that

$$\frac{dI_{ss}(V)}{dV} = 0 \quad \text{and} \quad \frac{d^2 I_{ss}(V)}{dV^2} = 0. \tag{19}$$

These two conditions determine several curves on the parameter space $(\bar{g}_{\text{Na}}, \bar{g}_{\text{KLT}})$, that limit the regions where a saddle-node bifurcation may or may not occur for a certain value of V.

Once we identified the region where there is only one fixed point we want to find conditions that guarantee that this fixed point remains stable for the whole range of values of I considered.

Consider the linearization of the system about the fixed point $(V, x_\infty(V))$, for $V \in (-100, 40)$ and let us denote A_V the associated Jacobian matrix. Recall that there is always a value of I for which $(V, x_\infty(V))$ is a fixed point of the system

(18). Asymptotic stability is guaranteed if all roots of the characteristic polynomial $P_A(\lambda) = \det(\lambda Id - A)$, satisfy $\Re(\lambda_i) < 0$. If we write this polynomial as

$$P_A(\lambda) = \lambda^n + q_{n-1}\lambda^{n-1} + \cdots + q_1\lambda + q_0,$$

we can use the Routh-Hurwitz criterion, that imposes conditions on the coefficients q_k of the characteristic polynomial, to guarantee asymptotic stability.

For $n = 2$ (S and D models), the fixed point is asymptotically stable if $q_0 > 0$ and $q_1 > 0$. Using equations (18) we have that

$$A_V = \begin{pmatrix} -\frac{\partial I_{ion}}{\partial V}(V,x) & -\frac{\partial I_{ion}}{\partial x}(V,x) \\ \frac{x'_\infty(V)}{\tau_x(V)} & -\frac{1}{\tau_x(V)} \end{pmatrix}_{|(V,x_\infty(V))}$$

and therefore

$$q_0 = \frac{dI_{ss}(V)}{dV},$$

and

$$q_1 = -\frac{\partial I_{ion}}{\partial V}(V, x_\infty(V)) - \frac{1}{\tau_x(V)}.$$

In order to guarantee that the fixed point does not change stability we need to impose that conditons $q_0 > 0$ and $q_1 > 0$ are satisfied for every $V \in (-100, 40)$. Notice that the first conditon guarantees the existence of a unique fixed point (no saddle-sode bifurcation occurs), while the second one guarantees that no Hopf bifurcation occurs.

For the S model, the black curve in Fig 3A left, which is given by the implicit equations (19), corresponds to the set of parameters $(\bar{g}_{\mathrm{Na}}, \bar{g}_{\mathrm{KLT}})$ for which there exist only one value of V for which $q_0 = 0$, while $q_0 > 0$ for the others. The curve separates the parameter space in two regions: the region where there exist an interval of values of V for which $q_0 < 0$, thus multiple steady states are possible (white area), and the region where $q_0 > 0$ for the whole range of values of V. In the red area, $q_0 > 0$ for all values of V, but there exist some values of $-100 < V < 40$ for which $q_1 \leq 0$, thus q_1 vanishes and a Hopf bifurcation occurs. In the grey area both $q_0, q_1 > 0$, thus the fixed point is stable for the whole range of voltages tested and no bifurcation occurs (type III excitability). The white area has both $q_0 < 0$ and $q_1 < 0$.

For the D model (see Fig 3A middle), in the grey area both $q_0, q_1 > 0$, thus the fixed point is stable for the whole range of voltages tested and no bifurcation occurs (type III excitability). In the red region, $q_0 > 0$ but there is at least one V for which $q_1 = 0$, thus a Hopf bifurcation occurs and the fixed point destabilizes.

For $n = 3$ (C model), the fixed point is asymptotically stable if $q_2 > 0, q_0 > 0$ and $q_1 q_2 > q_0$. In this case, the Jacobian matrix has the following form:

$$A_V = \begin{pmatrix} -\frac{\partial I_{ion}}{\partial V}(V,w,h) & -\frac{\partial I_{ion}}{\partial w}(V,w,h) & -\frac{\partial I_{ion}}{\partial h}(V,w,h) \\ \frac{w'_\infty(V)}{\tau_w(V)} & -\frac{1}{\tau_w(V)} & 0 \\ \frac{h'_\infty(V)}{\tau_h(V)} & 0 & -\frac{1}{\tau_h(V)} \end{pmatrix}_{|(V,w_\infty(V),h_\infty(V))}$$

If we write the matrix as:

$$A = \begin{pmatrix} a & b & c \\ d & e & 0 \\ g & 0 & l \end{pmatrix},$$

then the coefficients of the characteristic polynomial have the following form:

$$\begin{aligned} q_0 &= gce + bdl - ael \\ q_1 &= ae - bd + al + el - gc \\ q_2 &= -a - e - l. \end{aligned}$$

Notice that

$$\begin{aligned} q_0 &= \frac{\partial I_{ion}}{\partial V}(V, w_\infty(V), h_\infty(V)) \frac{1}{\tau_h(V)} \frac{1}{\tau_w(V)} + \frac{\partial I_{ion}}{\partial h}(V, w_\infty(V), h_\infty(V)) \frac{h'_\infty(V)}{\tau_h(V)\tau_w(V)} \\ &\quad + \frac{\partial I_{ion}}{\partial w}(V, w_\infty(V), h_\infty(V)) \frac{w'_\infty(V)}{\tau_w(V)\tau_h(V)} \\ &= \frac{1}{\tau_w(V)\tau_h(V)} \frac{dI_{ss}(V)}{dV}. \end{aligned}$$

Thus, since $\tau_w(V), \tau_h(V) > 0$, then $q_0 > 0$ as long as $dI_{ss}(V)/dV > 0$, which as we have seen guarantees the existence of a unique fixed point.

For the other two conditions we look for the regions on the parameter space $(\bar{g}_{\text{Na}}, \bar{g}_{\text{KLT}})$ for which conditions are satisfied for all values of V. We obtain the regions depicted in Fig 3A right.

As before the black curve is given by the implicit equations (19), and identifies the boundary that separates the region with only one steady state from those with multiple steady states. In the white region, $q_2 > 0$, $q_1 q_2 > q_0$ for all values of $-100 < V < 40$, but there exist some values of V for which $q_0 < 0$. Thus, multistability is possible. In the blue area there exist some values of V for which $q_2 < 0$ and $q_1 q_2 < q_0$, but still $q_0 > 0$. In the red area $q_0 > 0$ and $q_2 > 0$ for all $-100 < V < 40$, but there exist some values of V for which $q_1 q_2 < q_0$, thus $q_1 q_2$ changes sign and a Hopf bifurcation occurs. Indeed, the red region determines an area where there is destabilization of the fixed point through a Hopf bifurcation (for a certain range of voltages). In the grey area, $q_0 > 0, q_2 > 0$ and $q_1 q_2 > q_0$, thus the fixed point is stable for the whole range of voltages tested and no bifurcation occurs (type III excitability).

B Subtractive model with I_{KHT}

In order to develop a reduced S model, we first applied a similar strategy as for the D model and froze the inactivation h of sodium current at rest to avoid the contribution of divisive feedback, while keeping the maximal sodium conductance $\bar{g}_{\text{Na}}$ fixed. Unfortunately, the voltage for this reduced model fails to repolarize after spike onset when the input currents are high (Fig 12a, inset). We call this situation a *lockup.* The way to prevent lockup is decreasing the right peak of the V-nullcline (see Fig 12a). Along this paper, we were forced to reduce the value of $\bar{g}_{\text{Na}}$ to prevent this lockup state. In this section, we want to explain that other attemps to prevent this situation with other biological mechanisms were unsuccessful. Thus, we first tried by

adding back to the S model a fast activated high threshold potassium current I_{KHT}, similar to the one that was already present in the orginal RM03 model, see equation (2). In this case we consider

$$\begin{aligned} C\frac{dV}{dt} =& -2(\bar{g}_{\text{Na}} m_\infty(V)^3 h_0 (V - E_{\text{Na}}) + \bar{g}_{\text{KLT}} w^4 z_0 (V - E_{\text{K}}) \\ &+ g_{\text{l}}(V - E_{\text{l}})) + \bar{g}_{\text{KHT}} n (V - E_K) + I \\ \frac{dw}{dt} =& 3\frac{w_\infty(V) - w}{\tau_w(V)} \end{aligned} \tag{20}$$

with $n = n_\infty(V) = \left(1 + e^{-(v+15)/5}\right)^{-1}$ (see blue curve in Fig 12), $\bar{g}_{\text{Na}} = 1000$ and $h_0 = 0.1$ and the other parameters as in equation (3). We refer to this model as the instantaneous S+I_{KHT} model. Notice that I_{KHT} activates at a high voltage range ($V > -30$mV), and it brings down the right branch of the V-nullcline (Fig 12b). In order to bring down the right peak of the V-nullcline, we left shifted n_∞ by 8mV (Fig 12c) and incresed $\bar{g}_{\text{KHT}}$ from 150nS up to 600nS (Fig 12d). With increased $\bar{g}_{\text{KHT}}$, the right peak of the V-nullcline is decreased and the model becomes phasic again (Fig 12d). However, the spike amplitude is very small; its peak barely reaches -30mV (Fig 12d, inset).

Thus, instead of assuming instantaneous activation of I_{KHT} in equation (20), we consider a dynamic n:

$$\frac{dn}{dt} = 3\frac{n_\infty(V) - n}{\tau_n(V)}, \tag{21}$$

where

$$\tau_n = r_{\tau_n}\left(\frac{100}{4e^{(V+60)/32} + 5e^{-(V+60)/22)}} + 5\right), \tag{22}$$

and r_{τ_n} is a parameter that we will use to control the velocity of activation of I_{KHT}. We refer to this model as the dynamic S+I_{KHT} model. For $r_{\tau_n} = 1$ and $\bar{g}_{\text{KHT}}$=150nS, the model with dynamic I_{KHT} loses phasicness and shows periodic oscillations (limit cycle) when I is approximately in the range of 4.1 to 7nA (green dots in Fig 13a). Indeed, as I increases the fixed point of the system loses its stability through a Hopf bifurcation (HB). We increased $\bar{g}_{\text{KHT}}$ up to 600 nS, and the system still has a stable limit cycle (green dots in Fig 13b). The 2D bifurcation diagram for parameters $\bar{g}_{\text{KHT}}$ and I (Fig 13c) shows that increasing $\bar{g}_{\text{KHT}}$ does not prevent the system from oscillatory behavior. Next, we explored the role of the activation time constant of I_{KHT} by means of the parameter r_{τ_n}, and we computed the 2D bifurcation diagram for parameters r_{τ_n} and I (see Fig 13d). When $\bar{g}_{\text{KHT}} = 150$nS (black curves in Fig 13d), there exists a HB for the whole range of r_{τ_n}. However, when we increase $\bar{g}_{\text{KHT}}$ up to 600nS (red curves in Fig 13d), the HB disappears for very small r_{τ_n}. Notice that the left and right branches of the red curve connect to each other close to the x-axis. But here again for r_{τ_n} close to zero, spikes are too short. Thus, we did not find a satisfactory implementation that avoided the emergence of repetitive firing for strong inputs.

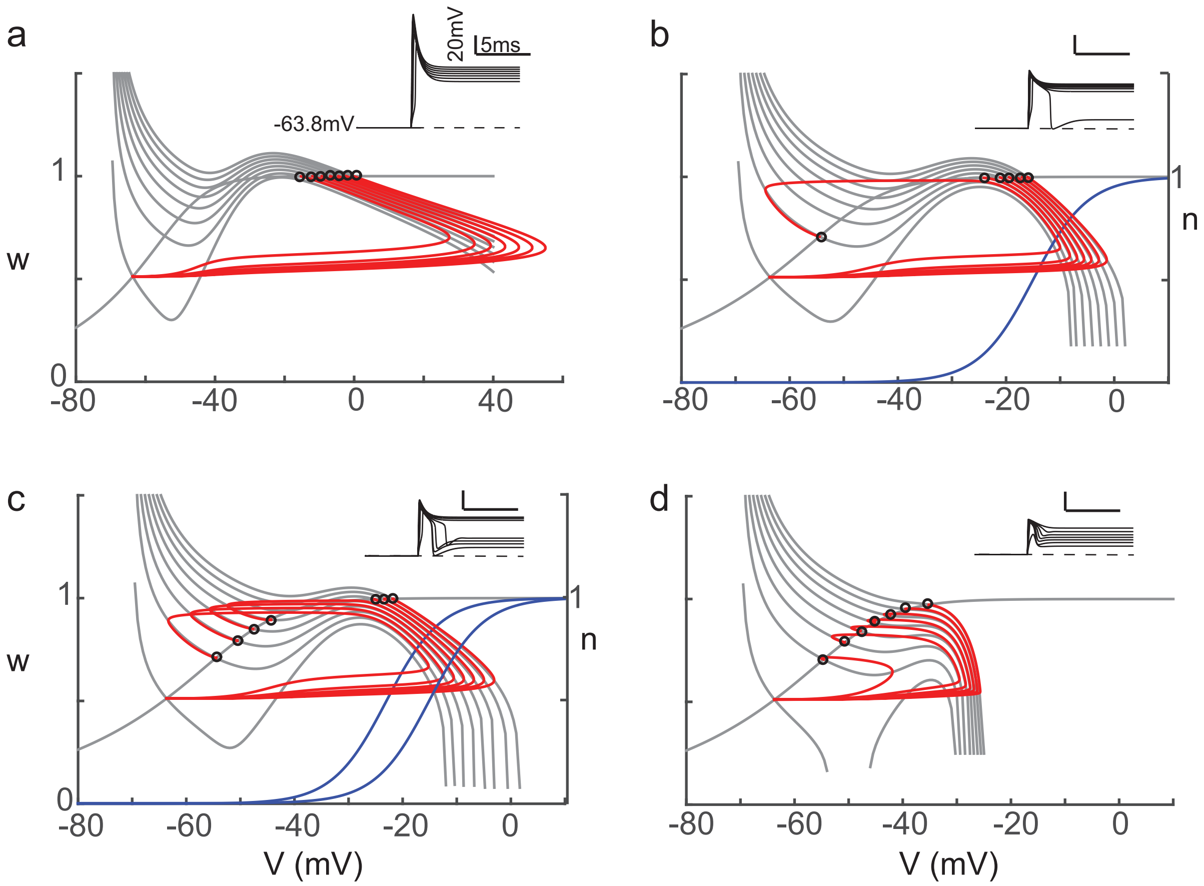

Figure 12: **Fast activation of high threshold potassium current in the instantaneous S+I_{KHT} model leads to small voltage peak**. (a-d) V-nullclines for different values of applied current I (0-7nA in steps of 1nA) (cubic-shaped grey curves) and w-nullcline (sigmoidal grey curve) for (a) the S model with $\bar{g}_{\mathrm{Na}} = 100$, (b) the instantaneous S+I_{KHT} model with $\bar{g}_{\mathrm{KHT}} = 150$nS, blue curve corresponds to $n_\infty(V)$, (c) the instantaneous S+I_{KHT} model, with $n_\infty(V)$ shifted leftwards by 8mV (blue sigmoidal curves illustrate the effects of the shift on $n_\infty(V)$) and $\bar{g}_{\mathrm{KHT}} = 150$nS, and (d) the instantaneous S+I_{KHT} model, with $n_\infty(V)$ shifted leftwards by 8mV (blue sigmoidal curve) and $\bar{g}_{\mathrm{KHT}} = 600$nS.

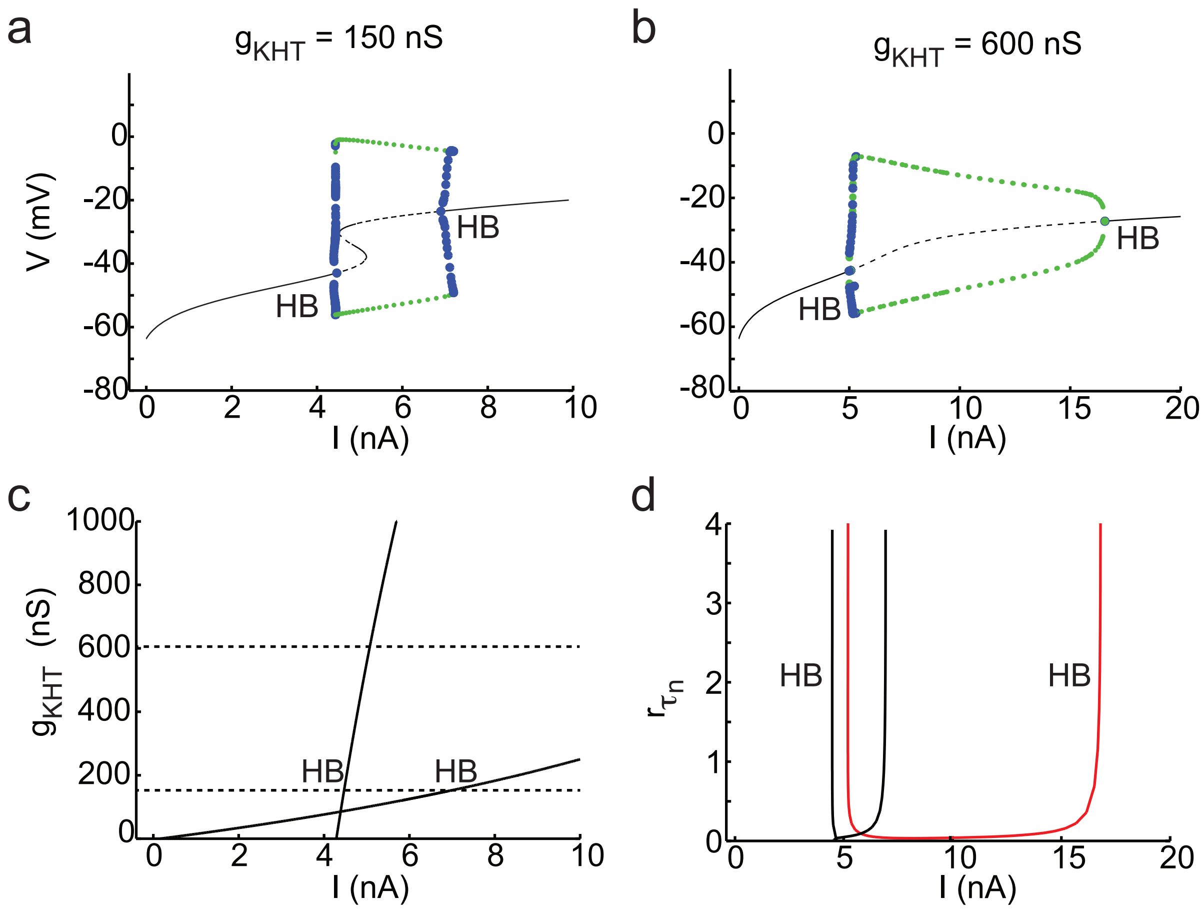

Figure 13: **Bifurcation diagrams for the dynamic S+I_{KHT} model for different values of $\bar{g}_{\mathrm{KHT}}$ and activation time constants.** (a, b) Bifurcation diagram of the S+I_{KHT} model for parameter I (input current) with (a) $\bar{g}_{\mathrm{KHT}}$=150nS and (b) $\bar{g}_{\mathrm{KHT}}$=600nS, and $r_{\tau_n} = 1$ (see equations (20)-(21)). Black solid and dashed curves correspond to stable and unstable fixed points, respectively. Green and blue dots correspond to maximal and minimal values of stable and unstable periodic orbits, respectively. Stable fixed point (black curve) loses its stability through a Hopf bifurcation (HB). (c) Two-parameter bifurcation diagram for parameters I and $\bar{g}_{\mathrm{KHT}}$, with $r_{\tau_n} = 1$ fixed. Dashed lines correspond to the bifuraction diagrams in panels (a-b). (d) Two-parameter bifurcation diagram for parameters I and r_{τ_n}, corresponding to different values of $\bar{g}_{\mathrm{KHT}}$ (black: $\bar{g}_{\mathrm{KHT}}$=150nS; red: $\bar{g}_{\mathrm{KHT}}$=600nS).
